# Reconsideration of Secure Communities rollout reveals preemptive local-federal cooperation in immigration enforcement

**DOI:** 10.1073/pnas.2510928123

**Published:** 2026-04-06

**Authors:** Cesar D. Vargas Nunez, Sakshina Bhatt, Basil F. Seif, Fernando S. Mendoza, David D. Laitin, Asad L. Asad

**Affiliations:** ^a^Immigration Policy Lab, Stanford University, Stanford, CA 94305; ^b^Department of Government, Claremont McKenna College, Claremont, CA 91711; ^c^Department of Pediatrics, School of Medicine, Stanford University, Stanford, CA 94305; ^d^Department of Political Science, Stanford University, Stanford, CA 94305; ^e^Department of Sociology, Stanford University, Stanford, CA 94305

**Keywords:** immigration enforcement, Secure Communities, deportation, policing

## Abstract

Qualitative studies on local police collaborations with federal immigration enforcement authorities reveal risks to the well-being of noncitizens, particularly the undocumented, and their families and communities. Yet statistical evidence of these policies’ effects is mixed. We propose that quantitative studies may misidentify the timing of when these policies begin disrupting immigrant communities by relying on a policy’s formal enactment date to indicate its activation. We test this proposal in the context of Secure Communities, a federal program with a staggered rollout that asked local police to detain noncitizens they arrested for possible transfer into Immigration and Customs Enforcement (ICE) custody and deportation. Individual states signed a Memorandum of Agreement (MOA) with the federal government as a framework for their county-by-county activation of Secure Communities. Counties were not required to activate immediately; their formal enactment frequently occurred later when prompted by ICE. We find that the date when a state signed an MOA consistently predicts a county’s increased probability of receiving ICE requests to hold noncitizens in detention, transferring detained noncitizens into ICE custody, and removing noncitizens from the country. This relationship operates most strongly in counties with preexisting enforcement infrastructure between local police and federal immigration authorities. By contrast, while we find that enactment dates are associated with increases in each outcome, pretreatment trends render these relationships statistically indeterminate. Our results highlight how multilayered relationships between local and federal authorities allow for policing to be used as a tool for facilitating the preemptive implementation of immigration enforcement across the country at the expense of noncitizens and their families and communities.

## Significance

Arrests by local police result in the transfer of many noncitizens into federal custody for possible deportation. From 2008 to 2013, the federal government rolled out the Secure Communities program to expand these local-federal collaborations. States signed memoranda of agreement (MOAs) outlining how counties would activate the program when prompted by Immigration and Customs Enforcement. While we find that enactment dates coincided with increased enforcement, we show that increases in detentions, transfers, and deportations began when states signed an MOA. Counties’ early participation, reflecting an overlapping infrastructure between local police and federal immigration officers, disrupted immigrant communities before any legal mandate. As local-federal collaborations expand, these results raise concerns about policing as a tool for preemptive immigration enforcement.

An estimated 11 million undocumented immigrants live in the United States (1). With greater vigor since the 1990s, the federal government has cultivated an interior immigration enforcement strategy that relies on collaborations with local police in counties nationwide to arrest, detain, and deport noncitizens, particularly the undocumented (2–4). This is known as policing as immigration enforcement (5). One consequence of relying on policing as a tool for immigration enforcement is that it renders the threat of deportation endemic to daily life for undocumented immigrants and their 4.4 million U.S. citizen children (6, 7). The threat of deportation may elevate risks such as undocumented immigrants and U.S. citizens’ increased psychological distress (8), as well as their estrangement from mainstream institutions that promote their families and communities’ health and well-being—including hospitals, the labor market, schools, and public assistance (9–14). Policing as immigration enforcement is not new (15). But the second Trump administration has renewed concerns about local-federal collaborations’ injurious potential following a flurry of executive orders and changes in enforcement practices that further leverage local police infrastructure for immigration enforcement (16). Therefore, a better understanding of whether and how local-federal collaborations alter the implementation of interior immigration enforcement across the country remains an important task.

Foundational to the federal government’s interior immigration enforcement strategy was Secure Communities (hereafter S-Comm) (5). Beginning in March 2008, with a staggered rollout across counties that ended in 2013 (17, 18), S-Comm built the technological infrastructure to expand local-federal collaborations for immigration enforcement.^*^ S-Comm asked local police to prolong the detention of undocumented immigrants they have arrested as part of their usual duties for possible transfer to Immigration and Customs Enforcement (ICE) custody and deportation. Each time local police arrested someone, they fingerprinted that person. Those fingerprints were processed by the State Identification Bureau [SIB; akin to a state-level Federal Bureau of Investigation (FBI)] and transmitted to the FBI. The FBI then ran those fingerprints through various federal criminal databases to ascertain whether the individual had an outstanding arrest warrant. S-Comm took advantage of this process by also having the FBI run the fingerprints through Department of Homeland Security (DHS) databases. If the database search flagged that person as a noncitizen who may be subject to deportation, ICE could issue a detainer request to the SIB; the SIB, in turn, passed this request to local police. The detainer asked, but did not require, participating police departments to hold the person in custody for an additional 48 h while ICE decided whether to assume custody. If ICE assumed custody, it could then initiate removal proceedings against the noncitizen, who could ultimately depart the country through one of several administrative mechanisms that functioned as removal (15).

S-Comm has been a point of contention in both scholarly and policy communities. From its inception, opponents lambasted S-Comm as “an extreme and draconian system that...will hurt the community by eroding trust in law enforcement, tearing families apart and shattering lives” (20). Yet nearly two decades since S-Comm’s enactment, the scholarly literature has accumulated mixed evidence of the program’s impact on the well-being of noncitizens and their families (9, 19, 21–24). Although qualitative studies based on small samples agree that S-Comm indeed has had these negative material and symbolic effects (5, 25–28), population-representative quantitative studies—despite strong theoretical and empirical reasons otherwise—have produced inconclusive results (21–24).

This study offers one explanation for the inconclusive quantitative results: the potential misidentification of when S-Comm activated. We posit that the date used to mark S-Comm as having been “activated” in a county matters for understanding that county’s experience with interior immigration enforcement. By activation, we refer to the date when a county begins to accept ICE’s requests to detain noncitizens for possible removal (“detainer requests”), often leading to noncitizens’ transfer into ICE custody (“transfers”) and eventual removal from the country (“removals”). When studies based on ethnographic observations and in-depth interviews describe S-Comm’s effects on individuals, families, and communities, they note an almost immediate perception among their study participants that their local context of immigration enforcement changed with the “start” of S-Comm (5, 26). But “start” is often used imprecisely, with it connoting at least two different meanings in the accumulating literature on S-Comm.^†^ On the one hand, the start may refer to a state’s signing date of a Memorandum of Agreement (MOA) with ICE. The MOA did not legally mandate that counties immediately participate in S-Comm; it was a framework to signal their future participation (29, 30). On the other hand, the start may refer to the enactment date, or when ICE designated a county as an “activated jurisdiction” for data-sharing under S-Comm in its administrative records (30).

Measuring the separate impacts of signing and enactment dates matters for both theoretical and empirical reasons. From a sociolegal perspective (31–34), we should expect different relationships between signing and enactment dates and interior immigration enforcement outcomes, including detainer requests, transfers, and removals. MOA signing dates did not mandate that states or their counties immediately expand policing as immigration enforcement. Rather, an MOA “on the books” signaled a state’s commitment to eventual participation. In prac- tice, however, many counties lacked live-scan technology capable of transmitting fingerprints to the FBI, and ICE initially lacked the capacity to screen all submissions received from the FBI (17, 18, 35). Moreover, even when a fingerprint submission resulted in a detainer request, the MOA did not require local jurisdictions to hold or transfer noncitizens into ICE custody. Taken together, these features suggest that signing alone should have had a weak relationship with interior immigration enforcement outcomes under S-Comm.

By contrast, enactment dates indicate S-Comm’s formal activation within a county. Given the same technological and capacity constraints, ICE conducted targeted outreach to local jurisdictions, “explaining when the jurisdiction is scheduled for activation” (30). This process was selective, prioritizing counties with large Latino populations along the US-Mexico border that were already inclined to cooperate with ICE (17, 18). Although localities could delay their activation, all counties were ultimately required to activate S-Comm by the end of 2013. Once enacted, ICE began issuing detainer requests to that county. To the extent that local officials interpreted activation as a federal directive to comply with at least some detainer requests, we would expect increases in not only detainers but also transfers and removals following enactment.

Yet, consistent with qualitative accounts of S-Comm (5, 25–28), what an MOA meant “in action” may have departed from its meaning “on the books.” Once a state signed an MOA, local and federal officials may have initiated cooperation immediately, well before a county’s formal activation. If so, signing—rather than enactment—would predict increases in detainers, transfers, and removals. Examining both signing and enactment dates, therefore, allows us to assess whether local-federal collaboration unfolds primarily through formal activation or through cooperation that begins in advance of any formal mandate.

Beyond measuring the separate impacts of signing and enactment dates on interior immigration enforcement resulting from S-Comm, we also consider one potential explanation for any observed relationship: preexisting partnerships between local police and federal immigration authorities. S-Comm was not the first program to leverage local-federal collaborations for immigration enforcement. To be sure, it was designed to supplant 287(g) agreements (28, 36). Authorized by Section 287(g) of the 1996 Illegal Immigration Reform and Immigrant Responsibility Act, these agreements deputized some local law enforcement officers to fulfill functions previously reserved for federal immigration authorities, such as interviewing individuals about their immigration status, checking DHS databases, and issuing detainer requests (28, 37). If signing and enactment dates have separate relationships with changes in interior immigration enforcement outcomes, it is plausible that prior local-federal partnerships via 287(g) play a role. Where prior 287(g) agreements existed, local police and federal immigration officers might have begun to implement S-Comm immediately upon the signing of an MOA despite no legal mandate to do so. Where these prior partnerships were unavailable, the enactment date might offer a better signal of S-Comm’s implementation. Given both local discretion and local-federal infrastructural constraints, only local jurisdictions with a prior 287(g) agreement might have been willing or prepared to activate S-Comm immediately upon their state signing an MOA.

This article examines trends in three consequential interior immigration enforcement outcomes-detainer requests, transfers into ICE custody, and removals-by the signing and enactment dates governing the rollout of S-Comm across U.S. counties. We find that signing dates consistently predict increases in each outcome. We also find that preexisting partnerships between local police and federal immigration authorities under 287(g) explain much of this result. By contrast, while we show that enactment dates are associated with increases in each outcome, pretreatment trends render these relationships statistically indeterminate. We conclude that the signing of an MOA served as a powerful signal for both local law enforcement and federal immigration authorities to carry out greater immigration enforcement before a legal mandate to do so, particularly in counties where federal–local collaborations were already institutionalized. We conclude with the potential implications of multilayered relations between local and federal authorities that allow for policing to be used as a tool for the preemptive implementation of immigration enforcement at the expense of noncitizens and their families.

## Materials and Methods

To measure the separate impacts of signing and enactment dates on increased interior immigration enforcement under S-Comm, we rely on multiple datasets. Data on whether and when a state signed an MOA with ICE come from Ariel R. White (38). Data on county enactment dates come from Marcella Alsan and Crystal S. Yang (35), as revised by Asad L. Asad and Livia Baer-Bositis (14), based on ICE reports listing activated counties. These data indicate the month in which a given county is treated, with treatment indicating that a county’s state has signed an MOA or a county has enacted S-Comm. We focus on the period beginning in January 2009 until December 2013; the first MOA was signed in 2009, and S-Comm was activated in all counties by December 2013. In analyses based on signing dates, all counties within a state receive the same treatment date because an MOA is permission for all counties within the state to activate S-Comm, though there is no legal mandate to do so within the MOA itself. In analyses based on enactment dates, counties within the same state may be treated at different times (17, 18). We visualize the staggered rollout of S-Comm based on signing and enactment dates in *SI Appendix*, Figs. S1–S3. In all analyses, we assign treatment based on the month and year of the signing or enactment date. On average, a state signed an MOA about 15.5 mo (sd: 9.27 mo) before all counties in the state enacted S-Comm. (See *SI Appendix*, Tables S1 and S2 for more details on signing dates, as well as the gap between signing and enactment dates. See also *SI Appendix*, Fig. S4.)

We merge these data sources with administrative data on three interior immigration enforcement outcomes: detainer requests, transfers into ICE custody, and removals. Data come from Syracuse University’s Transactional Records Access Clearinghouse (TRAC) (39). We focus on the approximately 1.23 million detainers that ICE issued under S-Comm across all counties in the continental United States between January 2009 and December 2013. ICE issued approximately 247,000 detainer requests per year, on average, during this period. For each measure, we drop any data without identifiable geographic information, a process we outline more fully in *SI Appendix*.

Although there are many potential impacts of interior immigration enforcement (40), we selected these three because they are among the most consequential (15). Since the 1990s, the rate of removals that began with an interior apprehension has proliferated (15). During the study period, more than half of all removals originating from within the United States started with an arrest by local police, who then transferred the individual to ICE custody (3). Understanding the institutional pathways between arrest and removal, therefore, helps clarify how this process evolved across counties and over time (41).

Our first outcome is a county-month-year count of detainer requests resulting from S-Comm. ICE issues a detainer request following a fingerprint submission from local police. Not all fingerprint submissions result in a detainer request, only those in which a database search identifies the fingerprints as belonging to someone whom ICE suspects is removable. We label each row in our data as a detainer request. For each county as defined by the FIPS code in the TRAC data, we total the number of detainers that ICE issued per month-year. We estimate an average of 6.6 (sd = 39.7) detainer requests per county-month-year across the study period.

Our second outcome is a county-month-year count of transfers of noncitizens from local police to ICE custody that resulted from S-Comm. Local police are not required to honor detainer requests, and ICE itself may not act on the detainer request. However, when local police and ICE do act on the detainer request, a noncitizen’s transfer into ICE custody implies that they remain on the path to removal. For each county, we measure transfers by calculating the total number of detainers that have a “booking date” indicating the month and year of their transfer to ICE custody. The TRAC data include identifiers that attribute a detainer to a given individual. Some individuals in the dataset have more than one detainer. Although double counting is not a threat to the validity of our first outcome analysis (e.g., the issuance of multiple detainers per person may imply separate police encounters), it may bias our results for transfers downward if we include multiple detainers per individual in our estimates. To avoid double counting, we randomly select a focal detainer for all individuals in the TRAC data with more than one detainer record. We estimate approximately 740,000 transfers across the study period, with an average of 3.9 (sd = 25.7) transfers per county-month-year.

Our final outcome of interest is a county-month-year count of the number of removals that resulted from S-Comm. A transfer into ICE custody does not necessarily imply that the noncitizen will depart or be removed from the country. If departure or removal occurs, it may be through “voluntary” or “involuntary” mechanisms (42). We consider any departure—voluntary or involuntary–as removal. To calculate removals, we count the total number of individuals with a “removal date,” an indication in the TRAC data that the arrested individual has departed the country. As with the transfer outcome, we randomly select one record for individuals who have more than one detainer. We estimate approximately 636,000 removals under S-Comm across the study period, with an average of 3.4 (sd = 21.3) removals per county-month-year. We consider this a lower-bound estimate of removals, as many detainers that resulted in transfers to ICE custody are right-censored; that is, they have pending decisions on their cases by the end of the study period.

Each outcome includes county-month-years that report zero detainers, transfers, and removals. To account for these zeroes, we binarize each outcome such that “1” indicates that a county has at least one detainer, transfer, or removal in a given month-year and “0” indicates otherwise (see *SI Appendix*, Figs. S5–S13 for visualizations of these variables’ distributions). We acknowledge that doing so comes at the cost of removing the intensity of these efforts across counties and time. Still, our results are robust when using a logarithmic (plus one) transformation for each outcome, as we show in *SI Appendix*, Figs. S14–S16.

To estimate the separate impacts of signing and enactment dates on each outcome, we leverage a difference-in-differences (DiD) design. This strategy allows us to compare treated and untreated counties by estimating what each outcome would have been in the treated counties had their state never signed an MOA agreeing to participate in S-Comm or had their county never enacted S-Comm. A key element of DiD is the assumption of parallel trends, where treated and untreated units share the same path pretreatment. We test for pretreatment trends using the F-test, which evaluates zero residual averages in the pretreatment period; a larger F-test suggests a better pretreatment trend fitting. We consider pretreatment trends in the presentation of our results below (*SI Appendix*, Fig. S17).

Our main analyses rely on a two-way fixed effects counterfactual (FEct) estimator (43). FEct is an extension of the traditional two-way fixed effects estimator that allows us to relax the assumption of constant treatment effects (44). Therefore, our results do not assume that the treatment–whether the signing or enactment date—yields the same magnitude across treated units. Allowing treatment effects to vary flexibly over time may reduce statistical power relative to more restrictive specifications, but it avoids imposing homogeneity assumptions that are unlikely to hold in a staggered and selectively implemented program like S-Comm. Results are also robust to using alternative estimators, such as interactive fixed effects counterfactual (IFEct) and matrix completion (MC) estimators (*SI Appendix*, Figs. S18 and S19).

The results below can be interpreted as the percentage point change in the probability of a detainer being issued, a transfer being effectuated, and/or a removal being completed following a state’s MOA signing or a county’s enactment of S-Comm. We present unadjusted models of these relationships. We also conducted analyses controlling for a range of characteristics associated with interior immigration enforcement (45), such as a county’s population composition (i.e., total, noncitizen, and Hispanic), the percentage of households below the poverty line, partisan vote shares, and crime rates. All results remain consistent with our main models (*SI Appendix*, Figs. S20–S25).

One potential threat to the validity of our results is selectivity in the rollout of S-Comm across counties and time. In the program’s early period, the federal government—not state or local jurisdictions—selected specific counties for activation (17, 18). Counties could not opt out once selected, though ICE sometimes agreed to delay activation dates (30). Still, when some jurisdictions sought to terminate their MOAs to “opt out” of S-Comm, ICE terminated all MOAs in August 2011 (i.e., seven months after the last MOA was signed across the study period) and emphasized that the program would be required of all jurisdictions eventually (17). However, before this termination, states could opt in to S-Comm through the signing of an MOA. The MOA granted a given state’s counties permission, but did not immediately require them, to begin participating in S-Comm.

To address potential concerns about S-Comm’s selective rollout, we identified counties that enacted S-Comm before their state had signed an MOA. Ninety counties met this criterion. Importantly, our main results are robust to excluding these counties from the analysis (*SI Appendix*, Figs. S26 and S27). For its part, we do not expect the termination of the MOAs to threaten our analysis; we compare counties whose states signed an MOA with counties whose states have not yet signed—or never did sign–an MOA. To the extent that we observe changes in the probability of each outcome following the signing of an MOA, it suggests that the MOA is itself consequential for the experience of interior immigration enforcement across counties.

Beyond measuring the separate impacts of signing or enactment dates on changes in interior immigration enforcement, we also consider whether preexisting partnerships between local police and federal immigration authorities under the 287(g) program account for our results. Data on 287(g) agreements come from Asad and Baer-Bositis (14). For each county-month-year, we code as “1” those jurisdictions that ever signed a 287(g) agreement before enrolling in S-Comm; about 24.3 percent (n = 755) of counties in our data signed a 287(g) agreement prior to enrolling in S-Comm. We include this indicator as a control variable and in split-sample regressions that replicate the main analyses separately for counties with and without prior 287(g) agreements. The former analysis evaluates whether any observed relationship between signing or enactment dates and interior immigration enforcement is explained by a prior 287(g) agreement; the latter evaluates whether this relationship varies by the presence or absence of a prior 287(g) agreement.

All analyses are conducted at the county level. This approach is appropriate for the analysis based on enactment dates since counties activated Secure Communities at different times. However, a potential challenge arises in the analysis based on signing dates, which exist only at the state level; all counties within a state, therefore, share the same signing date. In this context, SEs may need to be clustered at the state level to account for within-state correlation in outcomes (46). Results remain substantively similar in analyses that cluster by state, though CIs are, as expected, somewhat wider (*SI Appendix*, Figs. S28 and S29 and Table S5). We discuss the possible theoretical and methodological implications of clustering in *SI Appendix*.

## Results

**Fig. 1.**
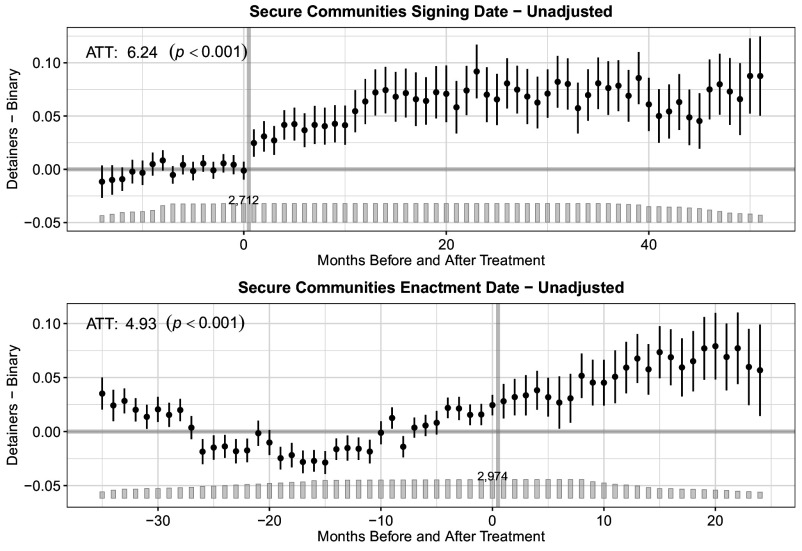
Effect of MOA signing or Secure Communities enactment on the percentage point change in the probability of a detainer request. The event study plots show the percentage point change in the probability of a detainer being issued across months before and after the signing of an MOA (*Top*) or the enactment of S-Comm (*Bottom*). The bar plot illustrates the number of treated units in each time period. The number atop the bar plot indicates the total treated units (i.e., counties). The average treatment effect on the treated (ATT) reveals the percentage point change in the probability of each outcome following treatment across all months in the posttreatment period.

**Fig. 2.**
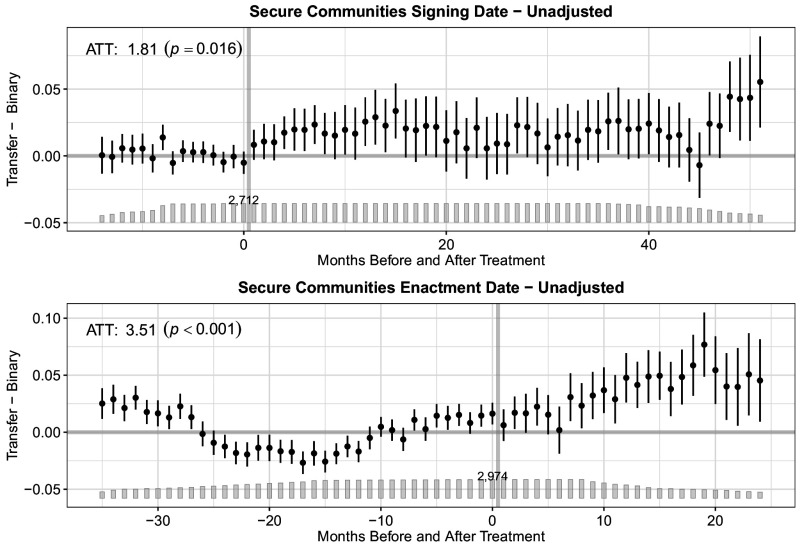
Effect of MOA signing or Secure Communities enactment on the percentage point change in the probability of ICE transfer. The event study plots show the percentage point change in the probability of a noncitizen being transferred into ICE custody across months before and after the signing of an MOA (*Top*) or the enactment of S-Comm (*Bottom*). The bar plot illustrates the number of treated units in each time period. The number atop the bar plot indicates the total treated units (i.e., counties). The average treatment effect on the treated (ATT) reveals the percentage point change in the probability of each outcome following treatment across all months in the posttreatment period.

**Fig. 3.**
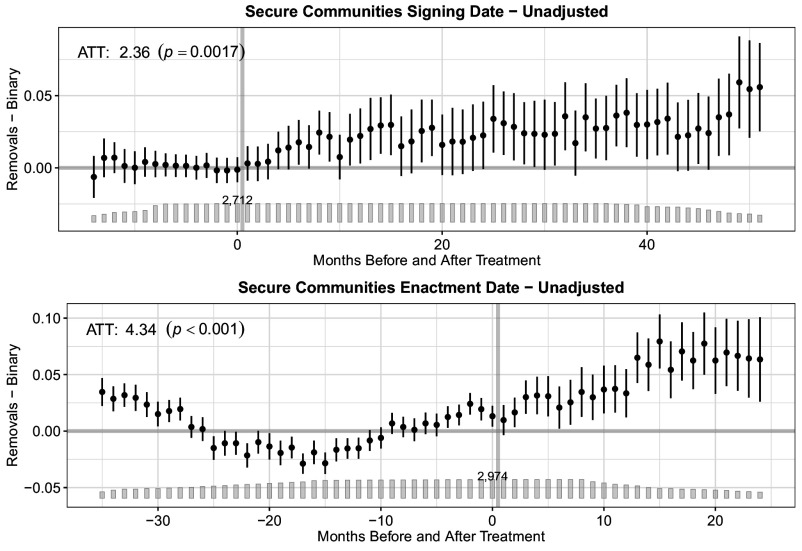
Effect of MOA signing or Secure Communities enactment on the percentage point change in the probability of removal.The event study plots show the percentage point change in the probability of a noncitizen being removed from the country across months before and after the signing of an MOA (*Top*) or the enactment of S-Comm (*Bottom*). The bar plot illustrates the number of treated units in each time period. The number atop the bar plot indicates the total treated units (i.e., counties). The average treatment effect on the treated (ATT) reveals the percentage point change in the probability of each outcome following treatment across all months in the posttreatment period.

Figs. 1–3 display our main results. All figures present event study plots showing each outcome (i.e., detainer requests, transfers, and removals) following a state’s signing of an MOA (*Top*) or county’s enactment of S-Comm (*Bottom*). The unadjusted coefficients represent month-to-month differences in the probability of each outcome between treated (i.e., those counties whose states signed an MOA or those counties that enacted S-Comm) and untreated (i.e., those counties whose states have not yet or never signed an MOA or that have not yet enacted S-Comm) counties before and after the focal treatment. The bar plot illustrates the number of treated units in each time period. The number atop the bar plot indicates the total number of counties under treatment in each month. While the month-to-month differences are instructive in evaluating trends in each outcome pre- and posttreatment, our primary interest is in the average treatment effect on the treated (ATT), which we display within each plot with its associated *P*-value. (See *SI Appendix*, Table S3 for the ATT with 95% CIs, and *SI Appendix*, Table S4 for fully adjusted results.) The ATT reveals the percentage point change in the probability of each outcome in the posttreatment period.

Fig. 1 shows results for detainer requests. Both signing and enactment dates appear to yield similar posttreatment month-to-month increases in the percentage point change in the probability of ICE issuing a detainer request. But the pretreatment data for enactment dates fail to reveal a clear effect (F test *P*-value = 0.000). Only for the signing date can we not attribute this increase to pretreatment trends (F test *P*-value = 0.155; see also *SI Appendix*, Figs. S20–S25). Following the signing of an MOA, we predict a 6.24 percentage point (*P* < 0.001; 95% CI: 4.60, 7.87) increase in the probability of ICE issuing a detainer request. The substantive interpretation of the ATT for the enactment dates analysis is similar but, given the presence of pretreatment trends, the effect is statistically indeterminate.

Fig. 2 shows results for transfers. As with detainer requests, only for the signing date can we not attribute observed increases in transfers to pretreatment trends (F test *P*-value = 0.204); the related F-test for the enactment date (F test *P*-value = 0.000) suggests pretreatment trends. Following the signing of an MOA, we find a 1.81 percentage point (*P*= 0.016; 95% CI: 0.33, 3.28) increase in the probability of an individual being transferred to ICE custody across the study period. As above, the substantive interpretation of the ATT for the enactment date analysis is similar but statistically indeterminate given the presence of pretreatment trends.

Finally, Fig. 3 shows results for removals. As with the two prior outcomes, only the signing date analyses evince a significant increase that cannot be attributed to pretreatment trends (F test *P*-value = 0.821). The F-test for the enactment date analysis again suggests the presence of pretreatment trends (F test *P*-value = 0.000). We find a 2.36 percentage point (*P*= 0.0017; 95% CI: 0.88, 3.84) increase in the probability of removal in counties after signing an MOA across the study period. The substantive interpretation of the ATT for the enactment dates analysis is, again, similar but statistically indeterminate.

What might explain the observed relationship between the signing dates of an MOA and interior immigration enforcement outcomes? Following existing research (17, 18), we consider whether a county’s preexisting relationship with federal immigration authorities accounts for this result. We do so in two ways: via the inclusion of a control variable in our models for whether a county had a 287(g) agreement with ICE before the signing of an MOA for S-Comm and via split-sample regressions distinguishing between counties with and without these prior agreements (see *SI Appendix*, Fig. S30 for the distribution of counties). We present the former results in *SI Appendix*, Figs. S20–S22; our results are robust to the inclusion of this control variable.

We present the results from the split-sample regressions in Table 1. The first column reproduces the ATT for our main analysis. The second and third columns present the results of our split-sample regressions. (See also *SI Appendix*, Table S6 for the fully adjusted estimates.) We find that the relationship between signing dates and the three interior immigration enforcement outcomes varies by a county’s prior history of participation in the 287(g) program. Among counties with a prior 287(g) agreement, we observe that the signing date of an MOA predicts an 11.5 percentage point (95% CI: 8.19, 14.8) increase in the probability of ICE issuing a detainer request, a 5.66 percentage point (95% CI: 2.42, 8.90) increase in the probability of a noncitizen being transferred into ICE custody, and a 5.54 percentage point (95% CI: 2.51, 8.57) increase in the probability of a noncitizen being removed from the country across the study period. The third column, which presents a similar analysis for counties without a prior 287(g) agreement, observes this dynamic only for the detainer outcome. In counties without a prior 287(g) agreement, the signing of an MOA predicts a 4.71 percentage point (95% CI: 2.72, 6.71) increase in the probability of ICE issuing a detainer request. The results for transfers and removals are indistinguishable from zero. Thus, while a prior 287(g) agreement does not appear to influence the DHS’ willingness to issue a detainer request, it does make a difference for whether the detainer leads to the eventual transfer of a noncitizen into ICE custody for subsequent removal. We further consider this result in *Discussion and Conclusion*.

**Table 1.** Unadjusted ATT (with 95% CIs) of signing date on percentage point change in the probability of detainer requests, transfers, and removals in counties with and without prior 287(g) agreements

	Overall	287(g) YES	287(g) NO
Detainers ATT	6.24	11.47	4.71
Detainers 95% CI Low	4.60	8.19	2.72
Detainers 95% CI High	7.87	14.75	6.71
Transfers ATT	1.81	5.66	0.54
Transfers 95% CI Low	0.33	2.42	−1.22
Transfers 95% CI High	3.28	8.90	2.30
Removals ATT	2.36	5.54	1.40
Removals 95% CI Low	0.88	2.51	−0.41
Removals 95% CI High	3.84	8.57	3.22

## Discussion and Conclusion

Our results suggest that the signing dates of MOAs related to S-Comm have measurable effects on consequential interior immigration enforcement outcomes, even though many counties had not yet formally enacted the program. We show that, upon a state’s signing of an MOA, its counties have not only an increased probability of receiving detainer requests from ICE but also cooperating with these requests by transferring noncitizens into ICE custody for eventual removal. Our analyses suggest that these effects operate in large part through preexisting relationships between local police and federal immigration authorities—namely, 287(g) agreements.

While our work establishes that MOA signing dates predict greater interior immigration enforcement in ways not attributable to pretreatment trends, additional work is needed to extend the analyses presented here. First, multimethod studies of what might be called the symbiotic relationship between local police and federal immigration authorities would help uncover the institutional mechanisms that allow policing as immigration enforcement to begin even absent a legal mandate to do so. To the extent that our results reflect the growing convergence of criminal and immigration law enforcement (19, 47), it bears identifying the mechanisms by which this convergence occurs on the ground. Second, systematic evaluation of policies that ostensibly temper the effects of immigration enforcement—namely, sanctuary policies that limit at least some police cooperation with ICE and that proliferated following the study period—is needed.^‡^ This evaluation should not presume sanctuary policies’ benevolence, given some research suggesting that these policies can reinforce local-federal collaborations by perpetuating the criminalization of noncitizens for low-level offenses (49, 50). Finally, research is needed to assess the possible differential effects of signing and enactment dates on noncitizens’ mental and physical well-being, and on that of their families and communities (9, 24, 51).

Results from this study nonetheless contribute to research on the interior structure and consequences of U.S. immigration enforcement. First, differentiating the agreement on the books from what it signals in action helps us better model how states and localities expanded policing as a tool of immigration enforcement (31–34). The agreement on the books creates a framework for a policy or program’s enactment; the agreement in action refers to the policy or program’s actual implementation. We find evidence that an agreement on the books can induce implementation before a program is formally activated. In the context of S-Comm, much of this owes to counties’ history of participation in the 287(g) program. While we do not find evidence that a prior 287(g) agreement explains the observed effects on detainer requests following the signing of an MOA, we find that it drives a large part of the main effects of the signing date on transfers and removals. This evidence supports, but does not directly confirm, our interpretation that a prior collaboration between local police and federal immigration authorities facilitated the transfer of noncitizens into ICE custody for eventual removal. This is not due to the higher concentration of noncitizens at risk of transfer and removal, as we see in *SI Appendix*, Table S4 and Figs. S20–S22 that results are robust to controlling for the population share of noncitizens in each county. We instead believe that this facilitation may occur for at least two reasons: Counties with a 287(g) agreement may enjoy a logistical advantage such that their prior experience prepared them to better collaborate with ICE (5), or they may be especially willing to participate in immigration policing (52). We cannot adjudicate these or other mechanisms here, and more than one explanation may be true. Still, our results validate claims described in ethnographic and interview-based research that local police may interpret the signing of an MOA as a powerful signal to begin participation in S-Comm before their counties’ formal activation (5, 25–27).

Although we have focused this article on S-Comm, it is not the only policy context in which we might observe the distinction between the law on the books and the law in action. Another example is Arizona’s Senate Bill 1070 (SB1070), which, among other provisions, would have required local police to determine a person’s legal status during a lawful traffic stop or when the officer had “reasonable suspicions” that the person lived in the country without authorization. An immediate legal challenge delayed and ultimately enjoined most of SB1070’s provisions. Still, research finds that the passage of the law, even absent its full enactment, was psychologically and physiologically harmful to Latino immigrants and their U.S.-born children (53). If the results of our study are any indication, it is possible that the passage of the law is enough to impel a change in local police and federal immigration authorities’ behavior on the ground. This logic may also extend to standalone federal or subnational initiatives bearing on immigration enforcement that end up challenged in court. For example, media reports suggest that law enforcement officers continue to enforce a Florida immigration law despite a federal judge’s prohibition to do so pending litigation (54). Beyond immigration enforcement, researchers studying other arenas of social life may also find the distinction between the law on the books and the law in action useful. For example, if a state passes a new minimum wage law, do employers immediately implement the law or wait until they are legally required to do so? The answer to this question likely has consequences for downstream analyses of the effects of the law’s passage and/or implementation.

Second, our results call attention to the importance of theorizing and empirically evaluating the mechanisms that underlie the effects of immigration enforcement policies on noncitizens and their families and communities. In the case of S-Comm, if the hypothesized mechanism operates through local changes to interior immigration enforcement practices, then the signing of the MOA constitutes a critical policy moment. This does not diminish the importance of enactment dates. Our results are compatible with the possibility that both signing and enactment dates shape interior immigration enforcement outcomes, even if only the former can be cleanly distinguished from pretreatment trends in our data. Enactment dates may operate through alternate pathways, such as shifts in media discourse, public awareness, or perceptions of interior immigration enforcement. We do not adjudicate among these mechanisms here. Still, these considerations underscore the need for future studies to disentangle the multiple pathways through which changes in interior immigration enforcement affect noncitizens and their families and communities (8, 19).

Finally, though interior immigration enforcement has a long history (15), the dynamics we outline have likely intensified under the second Trump administration. President Trump’s campaign for a second term emphasized the possibility of reaching as many as one million deportations per year. Within his first year in office, his administration has issued a flurry of executive orders and implemented several changes in ostensible pursuit of this goal. In January 2025, the administration issued an executive order that directed federal agencies to “[c]ooperat[e] fully with State and local law enforcement officials in enacting Federal-State partnerships to enforce Federal immigration priorities” (16). This executive order led to a modification of an injunction issued in February 2024 that prevented Texas state and local police from apprehending, arresting, and detaining undocumented immigrants. A related executive order invoked an emergency “mass influx” declaration for the first time (55), authorizing the DHS to sign agreements with state and local law enforcement agencies that deputize their officers as federal agents. The second Trump administration has also allowed ICE to lower detention standards to detain noncitizens in state and local jails and increased ICE’s use of automated license-plate-reader cameras for immigration enforcement (16). And, to continue building its interior immigration enforcement infrastructure, the federal government has begun recruiting agents from state and local police forces—something that local sheriffs have condemned (56). Our analysis suggests that this multilayered infrastructure risks disrupting immigrant communities before a legal mandate to do so (57). In so doing, the boundaries of criminal and immigration law become further blurred, to the detriment of noncitizens, their families, and their communities. Such detrimental collaborations are not inevitable even under the current structure of interior immigration enforcement; local police have discretion in whether and how much they facilitate the arrest, detention, and subsequent transfer of noncitizens into ICE custody for possible removal (58). To the extent that it is beneficial to circumscribe local-federal collaborations in immigration enforcement, greater scrutiny of these collaborations may be a worthwhile goal.

## Supplementary Material

Appendix 01 (PDF)

## Data Availability

The code and data necessary to replicate the results are available via GitHub (59).

## Footnotes

^*^Even when the Obama administration replaced S-Comm with the Priority Enforcement Program in 2013, it relied on the same data-sharing infrastructure established by S-Comm. The first Trump administration reinstated S-Comm, and the Biden administration discontinued it but left intact the data-sharing infrastructure (19).

^†^A third meaning may also be possible, which is when the federal government mandated a particular jurisdiction’s activation of Secure Communities, irrespective of the signing of an MOA (17, 18). We consider this meaning in *SI Appendix*, Figs. S26 and S27.

^‡^Scholars estimate that just 55 locales had enacted a sanctuary policy by December 2013 (48).
