# Supplementary material for: Reconsideration of Secure Communities rollout reveals preemptive local-federal cooperation in immigration enforcement
Source: Proc Natl Acad Sci U S A. 2026 Apr 6;123(15):e2510928123. doi: 10.1073/pnas.2510928123 (PMC13080013; doi:10.1073/pnas.2510928123)
Supplement: Supplementary file 1 — Appendix 01 (PDF) [file pnas.2510928123.sapp.pdf]

Supporting Information for

Reconsideration of Secure Communities Rollout Reveals Preemptive Local-Federal Cooperation in Immigration Enforcement

Cesar D. Vargas Nunez, Sakshina Bhatt, Basil F. Seif, Fernando S. Mendoza, David D. Laitin, Asad L. Asad

David D. Laitin; Asad L. Asad
E-mail: dlaitin@stanford.edu; asadasad@stanford.edu

This PDF file includes:

Supporting text
Figs. S1 to S31
Tables S1 to S6

Supporting Information Text

This SI proceeds as follows. First, it provides a summary of the evolution of Secure Communities in the U.S. during our study period. Second, it outlines key information needed to replicate our results, as well as data changes we included in our analyses. Third, it outlines a series of robustness checks, including different modeling specifications, use of alternate estimators, and changes in outcome variables. Fourth, we explore potential mechanisms that may influence the results. We conclude the appendix with information on the data used for the study and information on the process to merge the various datasets.

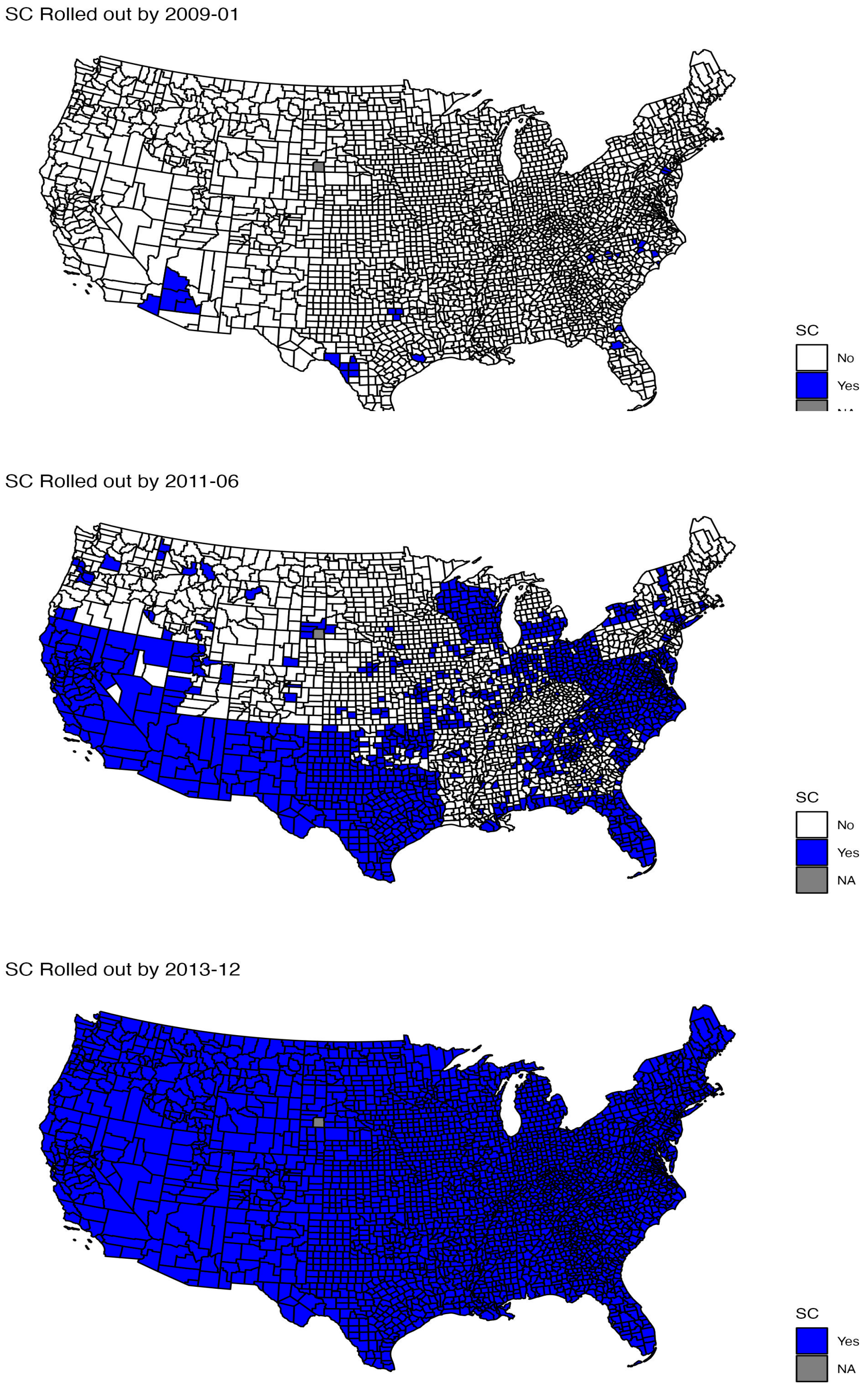

Fig. S1. Variation in Counties Enacting Secure Communities by January 2009, June 2011, and December 2013

Signing Dates. Most states in the continental U.S. signed, at some point, an MOA with ICE. We relied on White (2016) for all MOA signing dates. The only states to not sign an MOA are: AR, DC (considered a state for our purposes), MA, ME, MN, ND, NH, NJ, PA, VT, and WA. The signing dates for all other states are as follows:

State	Signing Date	State	Signing Date	State	Signing Date	State	Signing Date
AL	2010-11	IL	2009-11	NC	2009-10	SD	2010-06
AZ	2009-12	IN	2010-10	NE	2010-01	TN	2010-02
CA	2009-04	KS	2010-10	NM	2009-09	TX	2009-10
CO	2011-01	KY	2010-04	NV	2010-03	UT	2010-02
CT	2010-05	LA	2009-11	NY	2010-12	VA	2009-09
DE	2010-01	MD	2009-10	OH	2009-10	WI	2010-10
FL	2010-06	MI	2009-10	OK	2009-09	WV	2010-02
GA	2009-10	MO	2010-03	OR	2009-12	WY	2010-04
IA	2010-05	MS	2010-03	RI	2011-01		
ID	2010-04	MT	2010-02	SC	2010-08		

Table S1. Signing Dates Across States

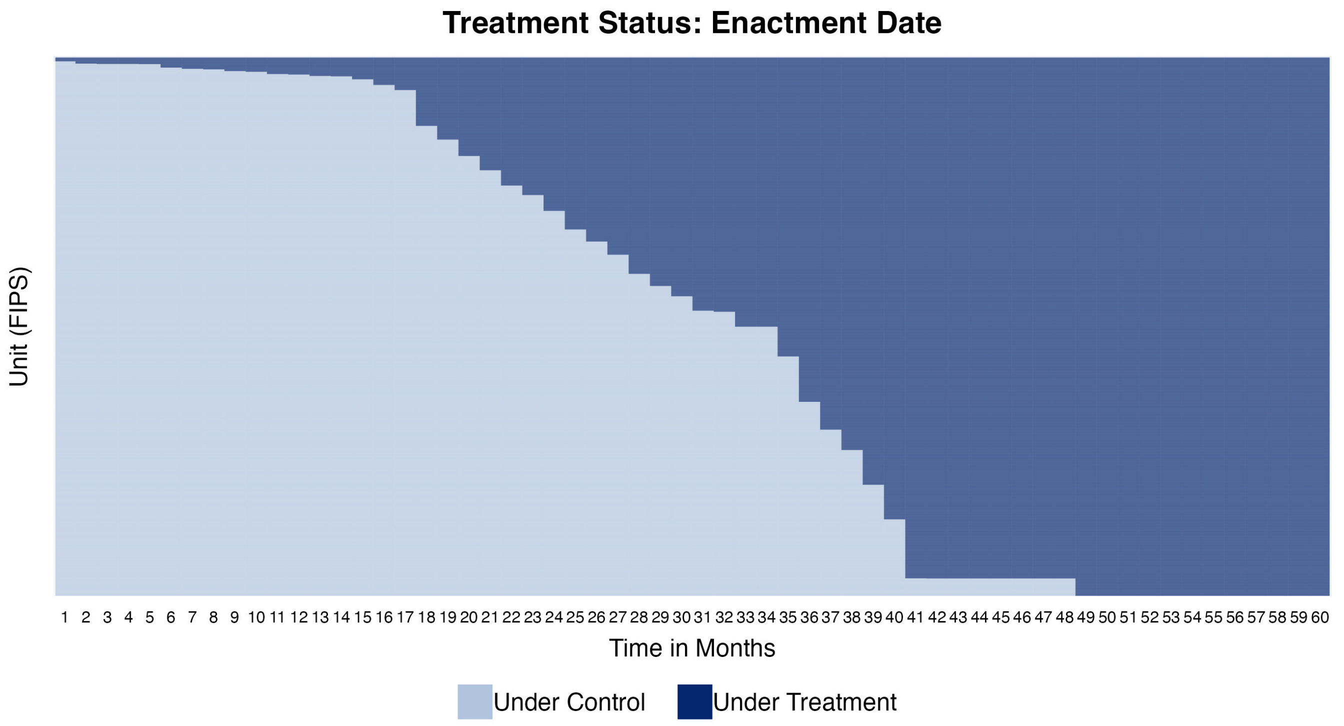

Fig. S2. Rollout of Enactment Treatment Status. The x-axis represents the timing of enactment by month with January 2009 as “1" and December 2013 as “60." The y-axis represents all counties in the continental United States.

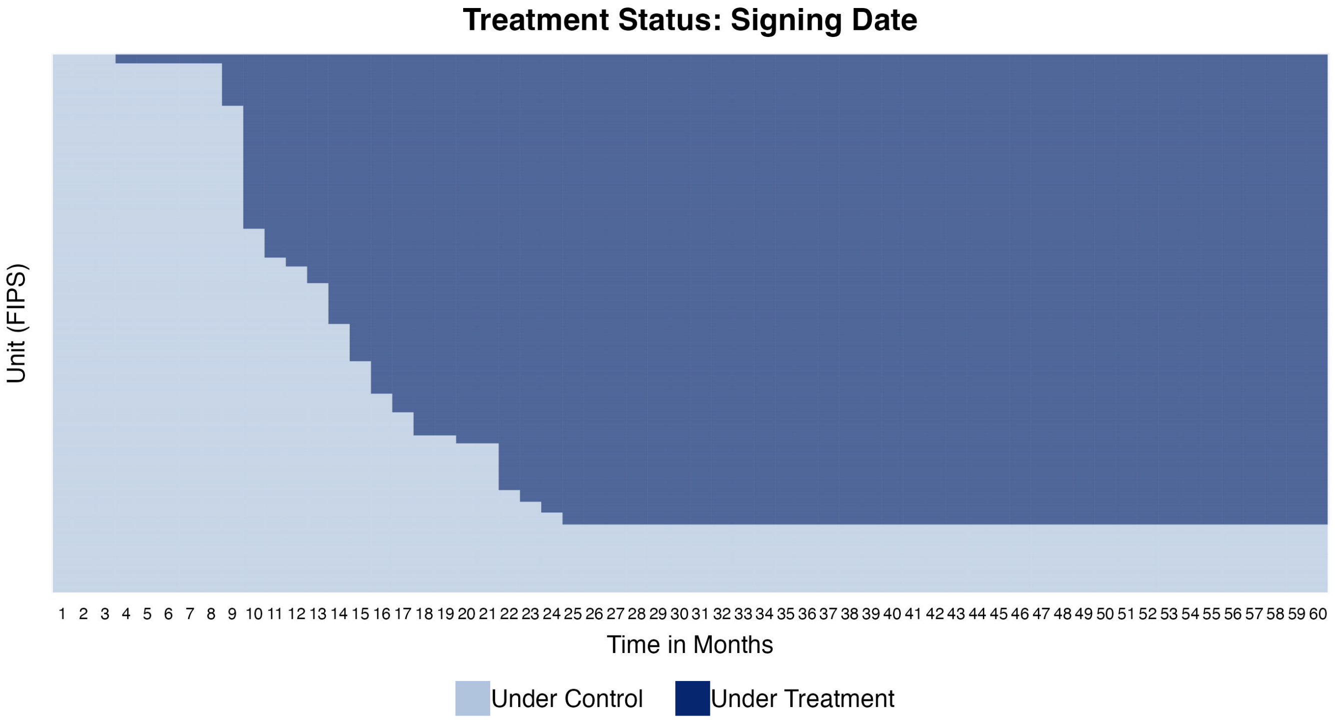

Fig. S3. Rollout of Signing Treatment Status. The x-axis represents the timing of MOA signing by month with January 2009 as "1" and December 2013 as "60." The y-axis represents all counties in the continental United States.

Steps for Calculating Counts of Outcome Variables. There are three situations that can lead ICE to issue more than one detainer for a given individual. First, an individual may have been apprehended on separate occasions. For instance, an individual may be arrested in time t, released from custody, and apprehended again in time $t+1$. In each of these situations, ICE may issue a separate detainer request.

Second, an individual may be transferred within or across police agencies. Officers may be asked to fingerprint all individuals assigned to their custody. If so, these multiple transfers within or across police agencies yield more than one set of identical fingerprints uploaded to the federal databases for a background check. ICE may issue a detainer request in each case.

Third, a detainer request provides ICE with 48 hours to assume custody of the individual. ICE, like any other agency, faces resource constraints. Issuing an additional detainer request may allow ICE the time to arrange for an officer to travel to the police agency.

Independent of the reason, multiple detainers for one individual may overestimate the number of transfers into ICE custody. Multiple detainers may also lead us to attribute a transfer from police custody to ICE to the wrong county.

We overcome these possible limitations with a two-pronged strategy. First, we identified the number of individuals with more than one detainer request. We use batch ID (provided by TRAC), unique case identifier (assigned by ICE), and booking date (date of detainer issuance or transfer date) to identify individuals with more than one detainer request. In total, we estimate about 22,000 cases of individuals having more than one detainer for the same arrest out of more than one million observations. Nearly 95 percent of these cases consisted of individuals having two detainer requests from ICE. Second, to address overcounting, we randomly selected one row for each individual with more than one detainer request. We ran the analyses on transfers and removals using the shortened dataset. We did not, however, change the dataset for the analyses on the number of detainer requests. After all, the increase in detainer requests reflects a conscious effort by ICE to increase its activity and enforcement.

Data Changes. We removed five FIPS (Federal Information Processing Series) codes from our analysis: 51780, 51560, 41006, 12025, and 30113. We removed 51780, 51560, and 12025 because they were merged into other FIPS codes before our study period. South Boston, VA (51780) was merged into Halifax County, VA. Clifton Forge, VA (51560) was merged into Alleghany County, VA. Dade County, FL (12025) was merged into Miami-Dade, FL. We removed the 30113 FIPS because it only covers the Yellowstone National Park area. Lastly, we removed the 41006 FIPS code because we could not find any information linking it to a specific geographic area. No observations in the detainers data were dropped due to these changes.

We also addressed two problems in the detainers data. First, we relabeled one observation whose FIPS code and state did not match. One observation in “Porter County" was mislabeled as part of Illinois. We addressed the misreporting by adding the correct Indiana state code. Second, we dropped two observations from “Decatur County" because the county does not exist in Illinois, and we could not determine the appropriate state.

Summary Characteristics. Overall, signing occurred prior to enactment. Figure S4 outlines the places where the enactment of Secure Communities occurred before or after the state signed an MOA. In total, there are 90 counties that enacted Secure Communities prior to their state's signing of an MOA. On average, state signing dates of an MOA occurred 15.5 months prior to enactment (SD = 9.27 months). The median gap between signing and enactment dates was 16 months, with the signing of an MOA occurring before enactment. Table S2 outlines the average, median, and SD gaps across states. Most of the states, except Florida, had an average gap with the state signing prior to the enactment of its counties.

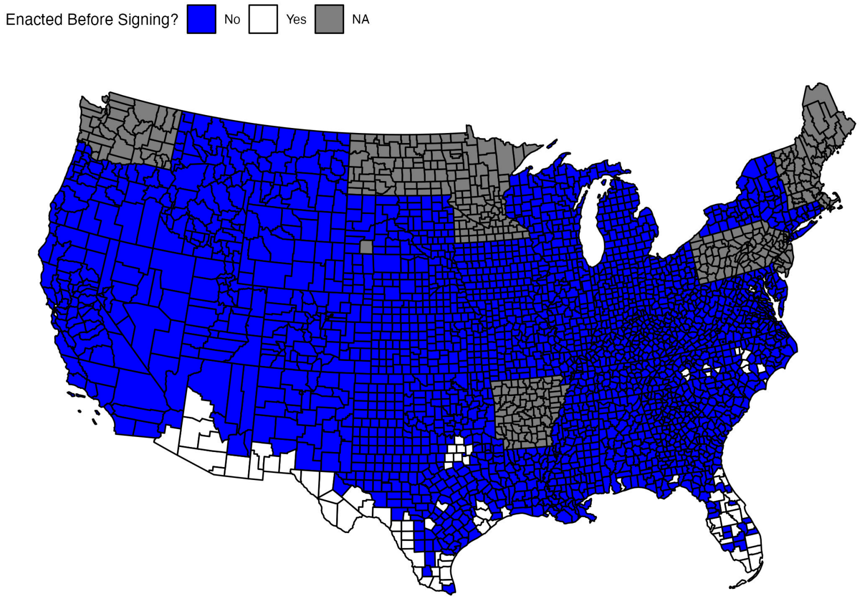

Fig. S4. Variation in Whether a County Enacted Secure Communities Before a State Signed an MOA, 2009-2013

state	mean difference (enacted - signing)	median months (enacted - signing)	SD
AL	-15.00	-8.00	9.94
AZ	-1.47	-7.00	8.58
CA	-15.52	-16.00	5.41
CO	-15.30	-16.00	3.17
CT	-18.50	-21.00	6.62
DE	-3.00	-3.00	0.00
FL	3.19	0.00	5.62
GA	-23.33	-26.00	5.11
IA	-18.63	-20.00	3.63
ID	-17.52	-19.00	3.92
IL	-30.52	-38.00	12.91
IN	-9.99	-8.00	3.50
KS	-12.90	-14.00	2.91
KY	-22.86	-23.00	1.55
LA	-26.22	-30.00	7.51
MD	-15.46	-16.00	5.81
MI	-20.86	-23.00	3.78
MO	-16.56	-20.00	4.22
MS	-16.90	-18.00	4.19
MT	-24.89	-26.00	4.66
NC	-9.40	-12.00	7.73
NE	-23.98	-26.00	5.32
NM	-12.09	-14.00	5.92
NV	-12.06	-10.00	7.14
NY	-10.24	-11.50	6.84
OH	-16.57	-17.00	3.15
OK	-19.87	-20.00	5.67
OR	-18.86	-21.00	4.90
RI	-2.00	-2.00	0.00
SC	-7.52	-8.00	4.00
SD	-20.12	-22.00	5.95
TN	-19.89	-23.00	5.92
TX	-7.04	-9.00	5.71
UT	-13.90	-22.00	8.99
VA	-8.29	-9.00	2.28
WI	-3.00	-3.00	0.00
WV	-8.00	-8.00	0.00
WY	-24.30	-25.00	3.26

Table S2. Mean and Median Differences in the Signing and Enactment of Secure Communities across States, 2009-2013

Data Spread. Detainers

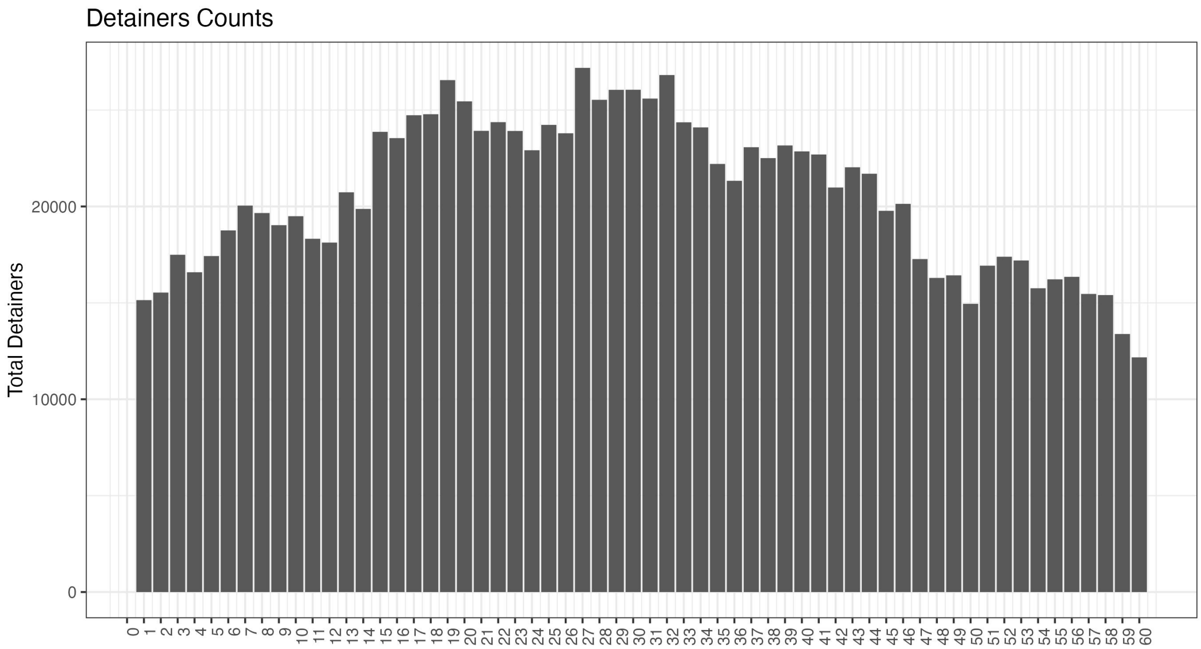

Fig. S5. Detainer Counts Across Time. Each month in the x-axis represents a month, with January 2009 = “1" and December 2013 = “60."

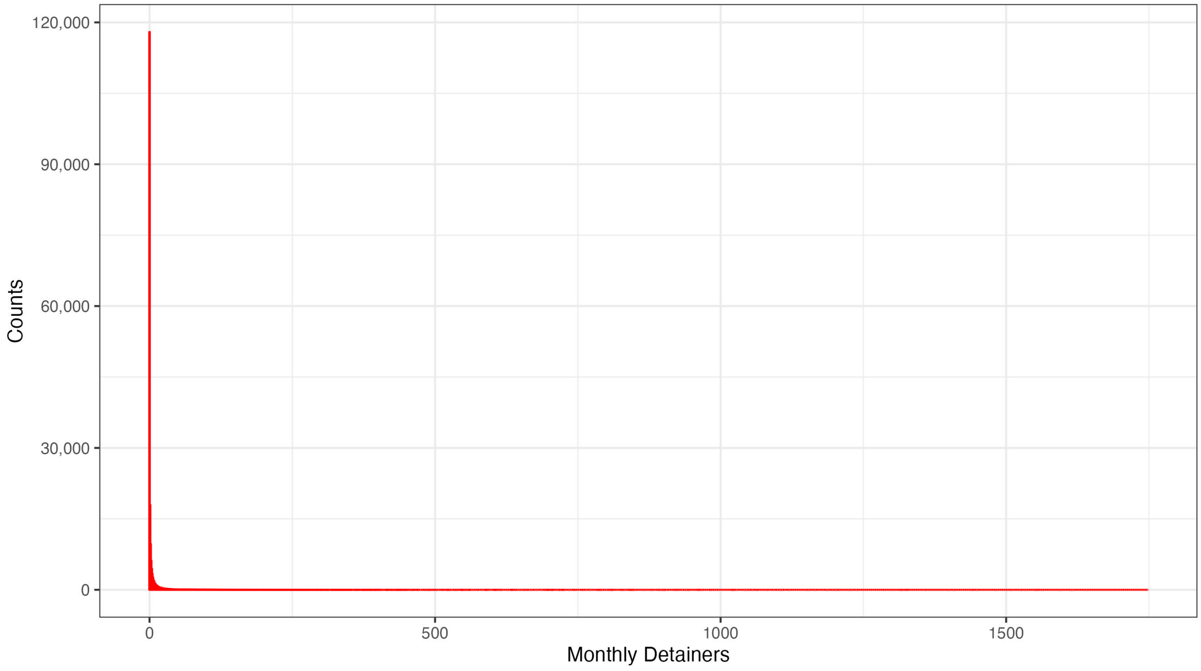

Fig. S6. Histogram of Monthly Detainer Counts

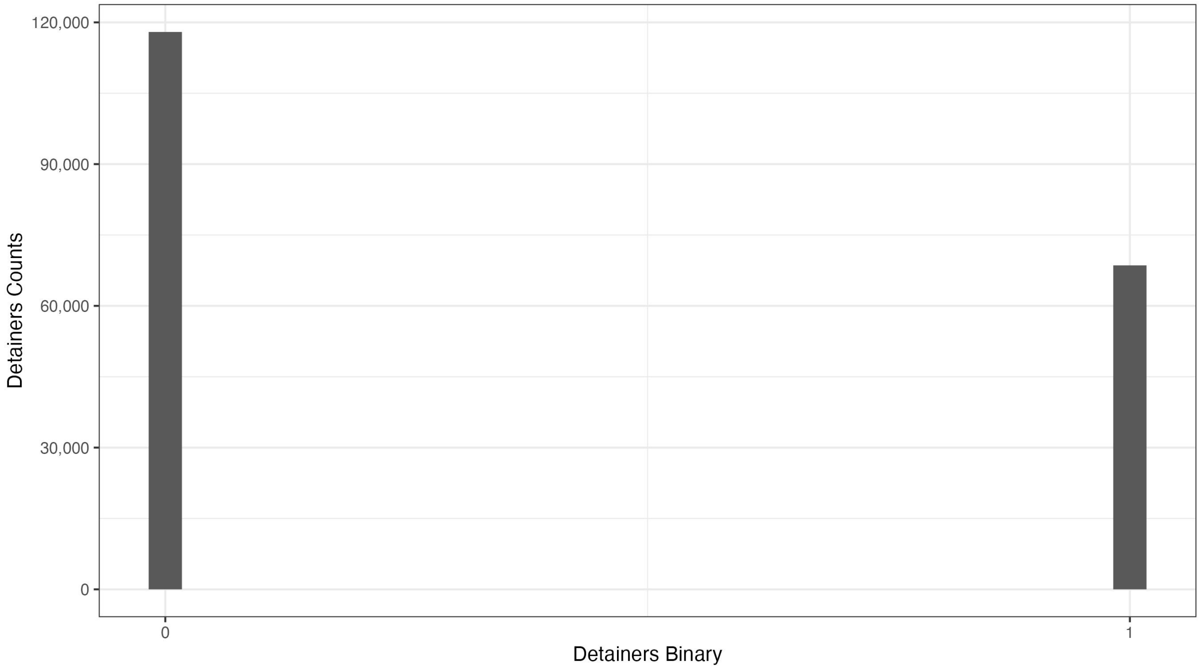

Fig. S7. Histogram of Monthly Detainer Counts with Binary Transformation

Transfers

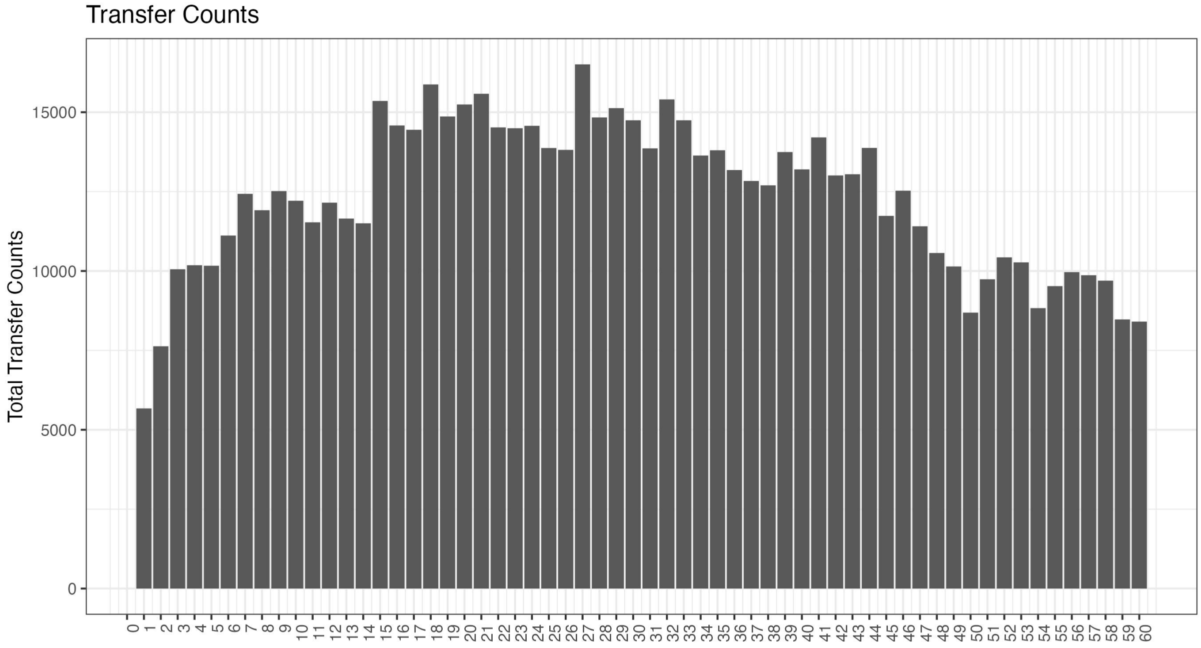

Fig. S8. Transfers Counts Across Time. Each Month in the x-axis Represents a Month with January 2009 = 1 and December 2013 = 60.

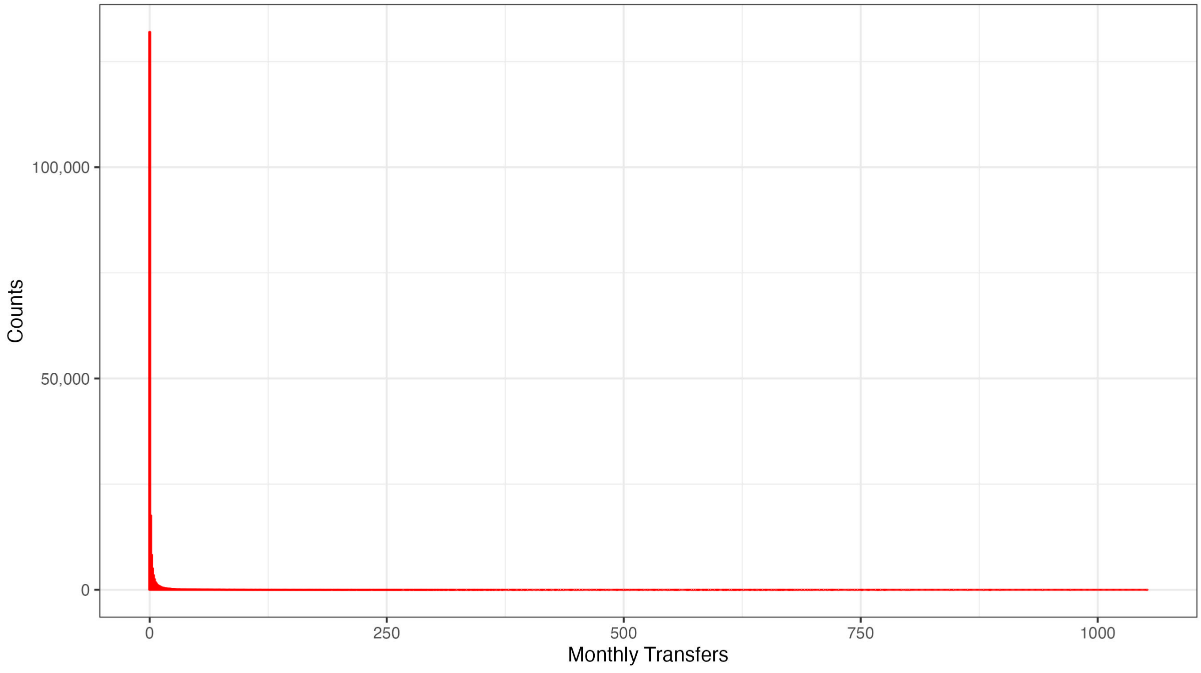

Fig. S9. Histogram of Monthly Transfer Counts

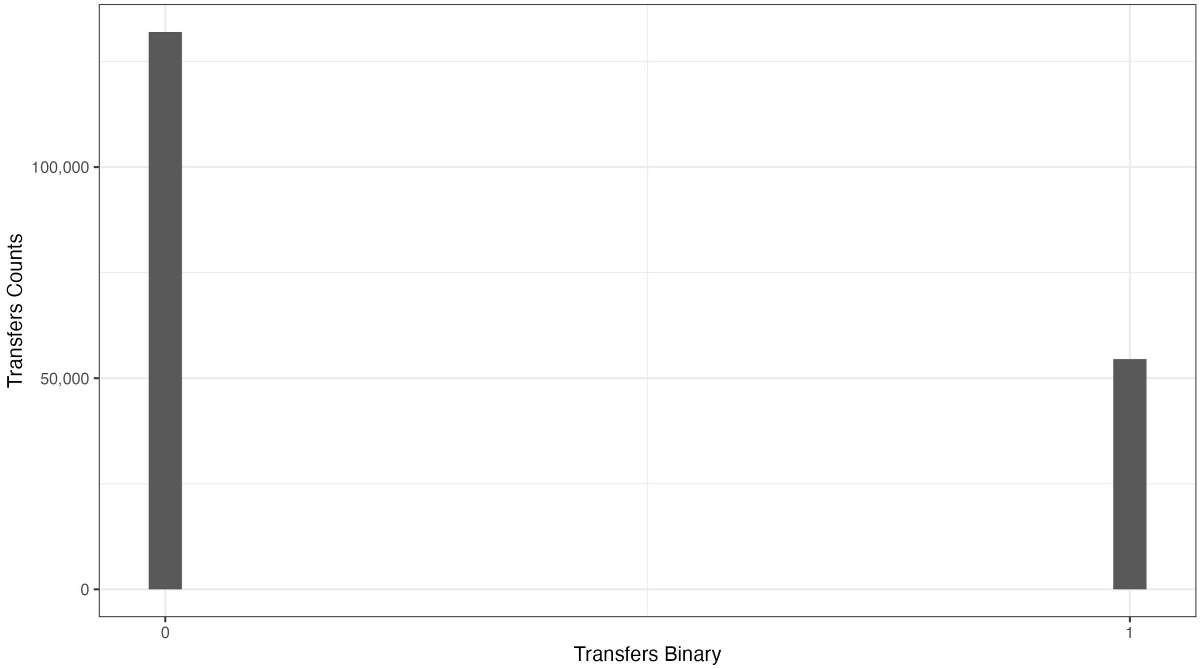

Fig. S10. Histogram of Monthly Transfer Counts with Binary Transformation

Removals

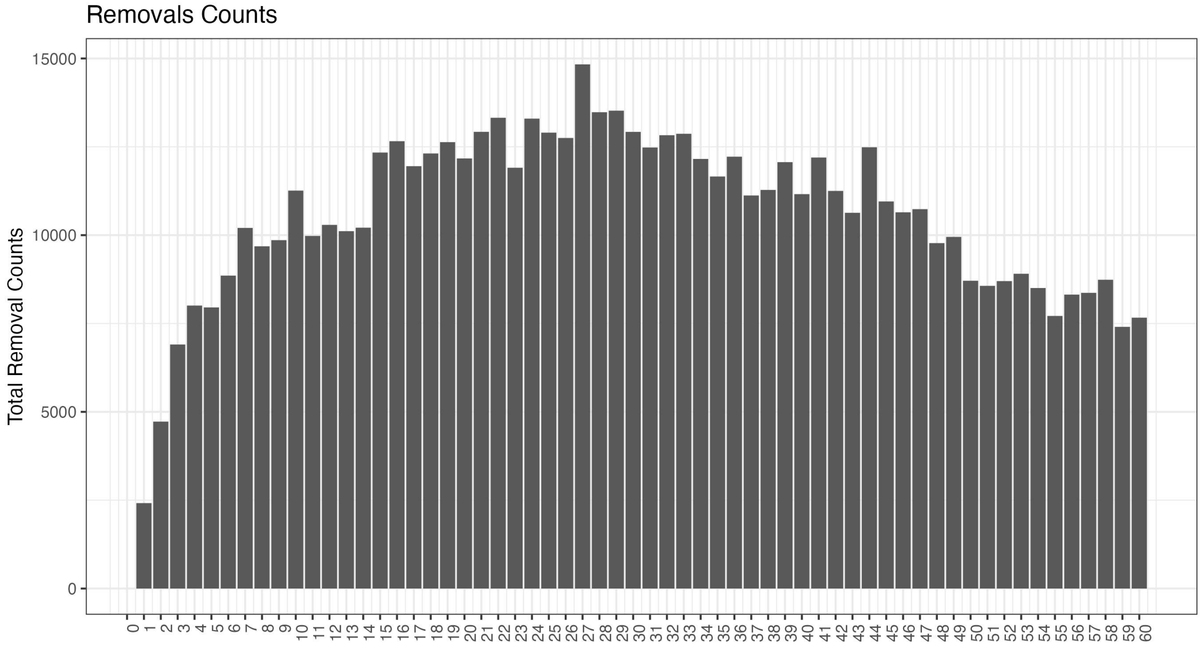

Fig. S11. Removals Counts Across Time. Each Month in the x-axis Represents a Month with January 2009 = 1 and December 2013 = 60.

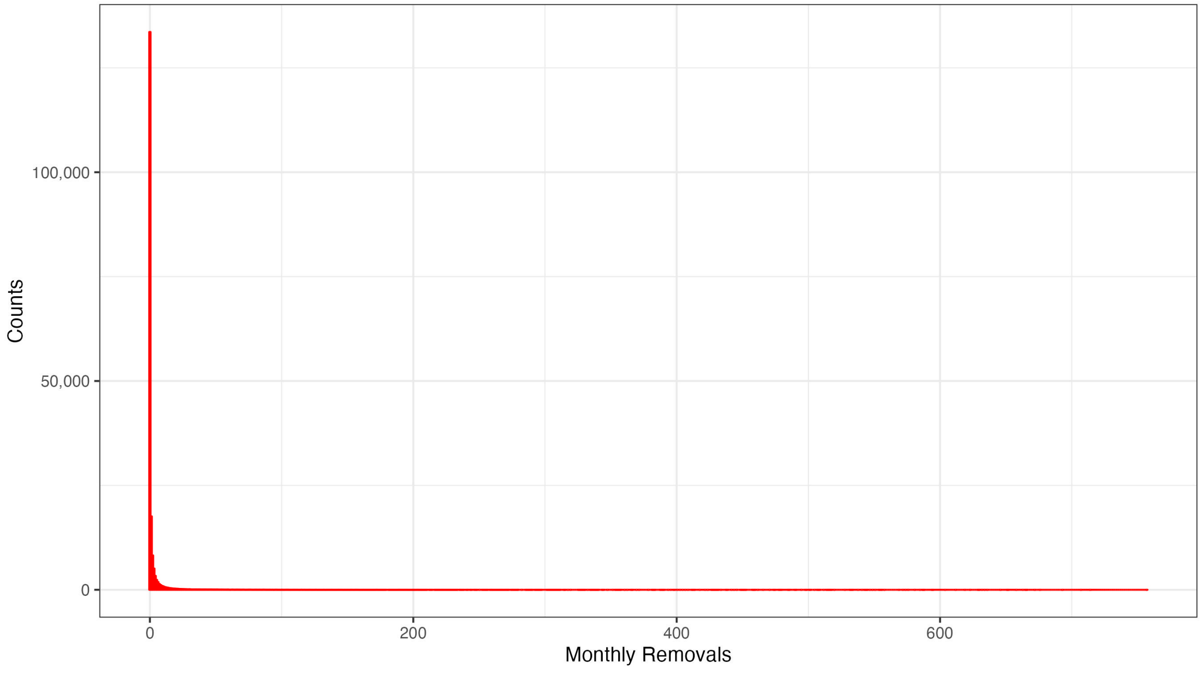

Fig. S12. Histogram of Monthly Removals Counts

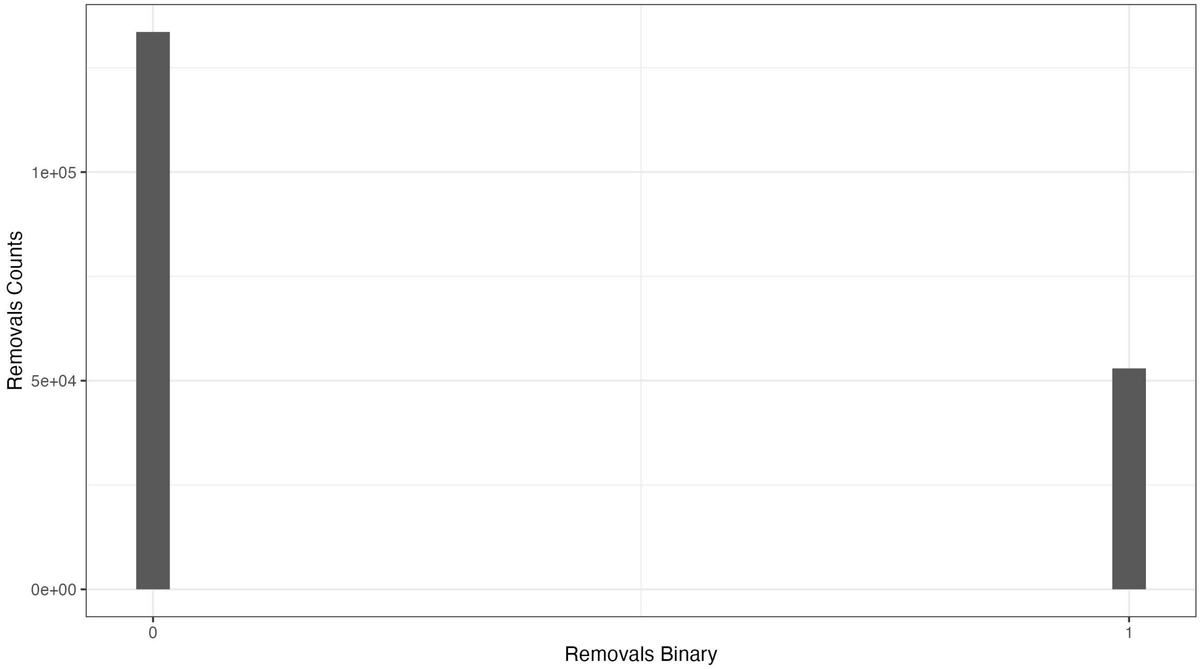

Fig. S13. Histogram of Monthly Removals Counts with Binary Transformation

Statistical Analyses. We relied on a Difference-in-Differences design to investigate the effect of Secure Communities on immigration enforcement in a plausibly-causal framework. We relied primarily on the following generalized linear model:

$$Y_{i,j} = \alpha_i + \omega_j + \beta_D \Gamma_{i,j} + \epsilon_{i,j} \quad [1]$$

where $Y_{i,j}$ is an indicator for counts in month i and county j; α_i is an indicator for time fixed effects; ω_j is an indicator for county fixed effects, β_D is the coefficient of interest reflecting when the treatment turned on for county j in month i; $\epsilon_{i,j}$ is an error term.

Outcomes: We focused on three main outcomes: detainers, transfers, and removals. We relied on data obtained via TRAC at Syracuse University for all outcomes. We excluded any rows without identifiable geographic information. Missing rates varied but ranged from a peak of 36,000 in 2009, to a low of around 8,000 in 2011. We present below our coding process for each outcome variable and other relevant information. We also note that the y-axis in our figures is not always the same given the differences in the number of detainers, transfers, and removals. Readers should be mindful of the shift in scale.

- Detainers - we labeled each row in our data as an ICE-issued detainer request. Next, we added all counts per month for each county (identified using the FIPS code). This number became our outcome of interest. For the results in the main text, we labeled each county as “0" in months without any detainers and “1" in any months with one or more detainer.
- Transfers - we counted the total number of rows of individuals who had a “booking date," which indicates the month when ICE assumed their custody. While nearly all cases had individuals with one detainer issued on their case, some had more than one. To address double counting, we randomized and only counted the selected row for all individuals with n+1 detainer requests issued on their case. The final outcome of interest was the monthly sum of all individuals transferred into ICE custody per county. For the results in the main text, we labeled each county as “0" in months without any transfers and “1" in months with 1 (or more) transfers.
- Removals - we counted the total rows of individuals with a “removal date" indicating that the arrested individual has been removed from the country. Although the data also include information outlining whether the departure was voluntary or by force, we did not use this distinction in our analyses. Whether voluntary or by force, both removals begin with an involuntary apprehension that results in their departure from the country. Similar to the transfer outcome, we had some cases in which individuals had more than one detainer issued on their case. To address overcounting, we randomized the row for all individuals with n+1 detainer requests issued on their case and only counted the one selected. Our final outcome was the monthly sum of all individuals removed from the country per county. For the results in the main text, we labeled each county as “0" in months without any removals and “1" in months with 1 (or more) removals.

Alternate Outcome Specifications. Our data present some modeling challenges. Because our data focus on monthly counts of detainers, transfers, and removals across counties, there are many zeroes. As seen in Figures S6 - S12, there is also large dispersion of monthly counts across counties. Any data transformation comes with tradeoffs. In the main text, we showed results from transforming the counts into a binary outcome, indicating the presence of 0 or 1 or more detainer, transfer, or removal counts per month. The cost of this transformation is that it removes the possible intensity of the treatment across county-months.

We addressed the prevalence of zeroes by leveraging another transformation and taking the log (plus one) of the outcome. The main benefit is that doing so preserves (some) intensity of the treatment. But logging outcome variables also faces significant drawbacks, such as introducing bias and loss of information (Chen and Roth 2024). Doing so also requires us to assume the effect is multiplicative rather than additive.

Figures S14 - S16 show the unadjusted results after transforming the outcome into log plus one for detainers, transfers, and removals. The results in S14 show that the signing of an MOA was associated with a significant increase of 13.1 percentage point in detainers issued ($p < .01$, 95% CI: 9.49, 16.7). Similarly, Figure S15 shows that increased detainers also translated into a significant increase of 5.2 percentage points in the number of individuals transferred into ICE ($p < .01$, 95% CI: 2.3, 8). Finally, Figure S16 shows that the signing triggered a significant increase of 5.3 percentage points in the number of individuals removed from the country ($p < .01$, 95% CI: 2.2, 8.3).

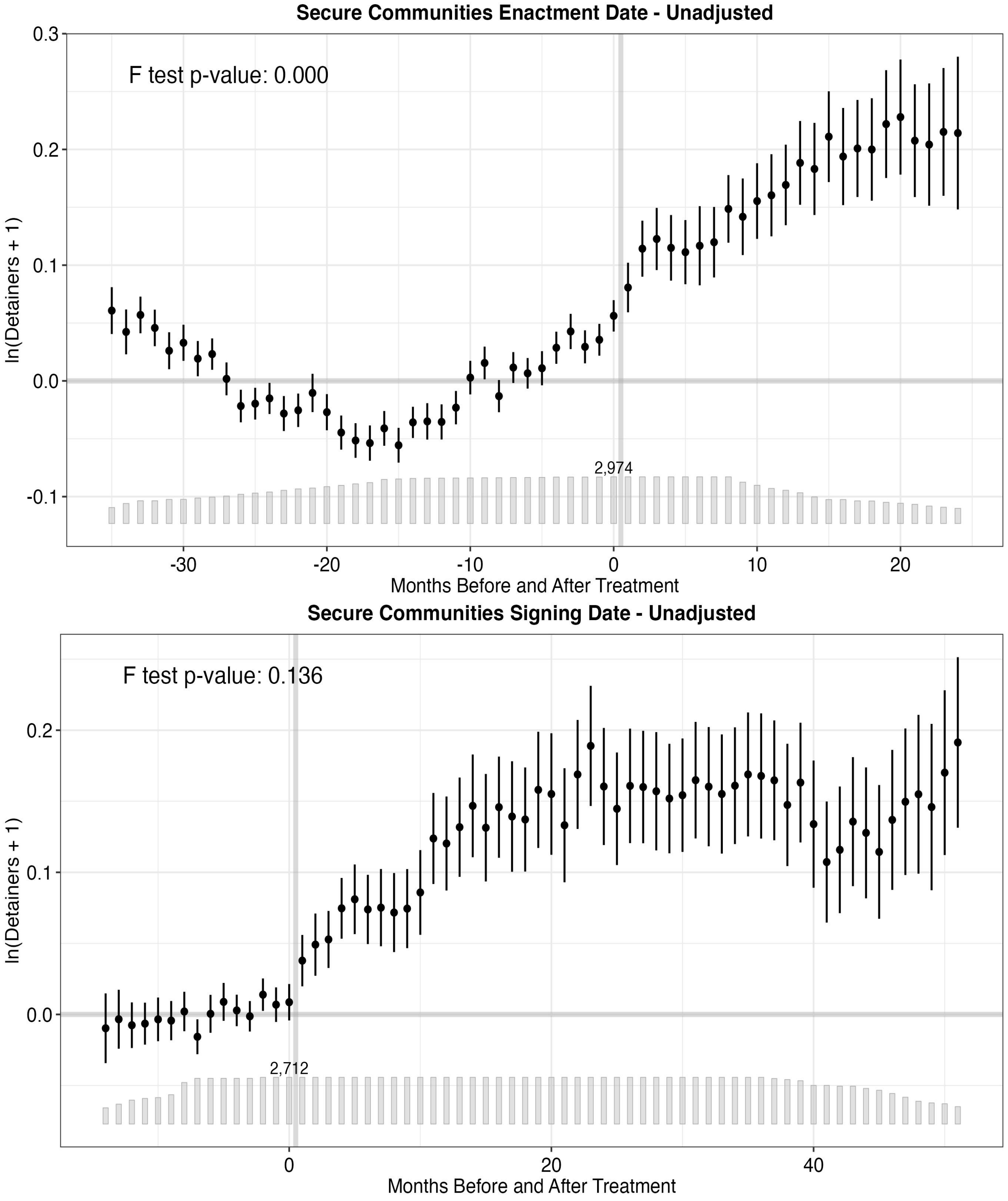

Fig. S14. Impact of Secure Communities Enactment (top) and Signing (bottom) on Detainers issued by ICE. The number within the figure indicates the total treated units (counties). The F-test evaluates zero residual averages in the pre-treatment period. A larger F-test p-value suggests a better pre-trend fitting.

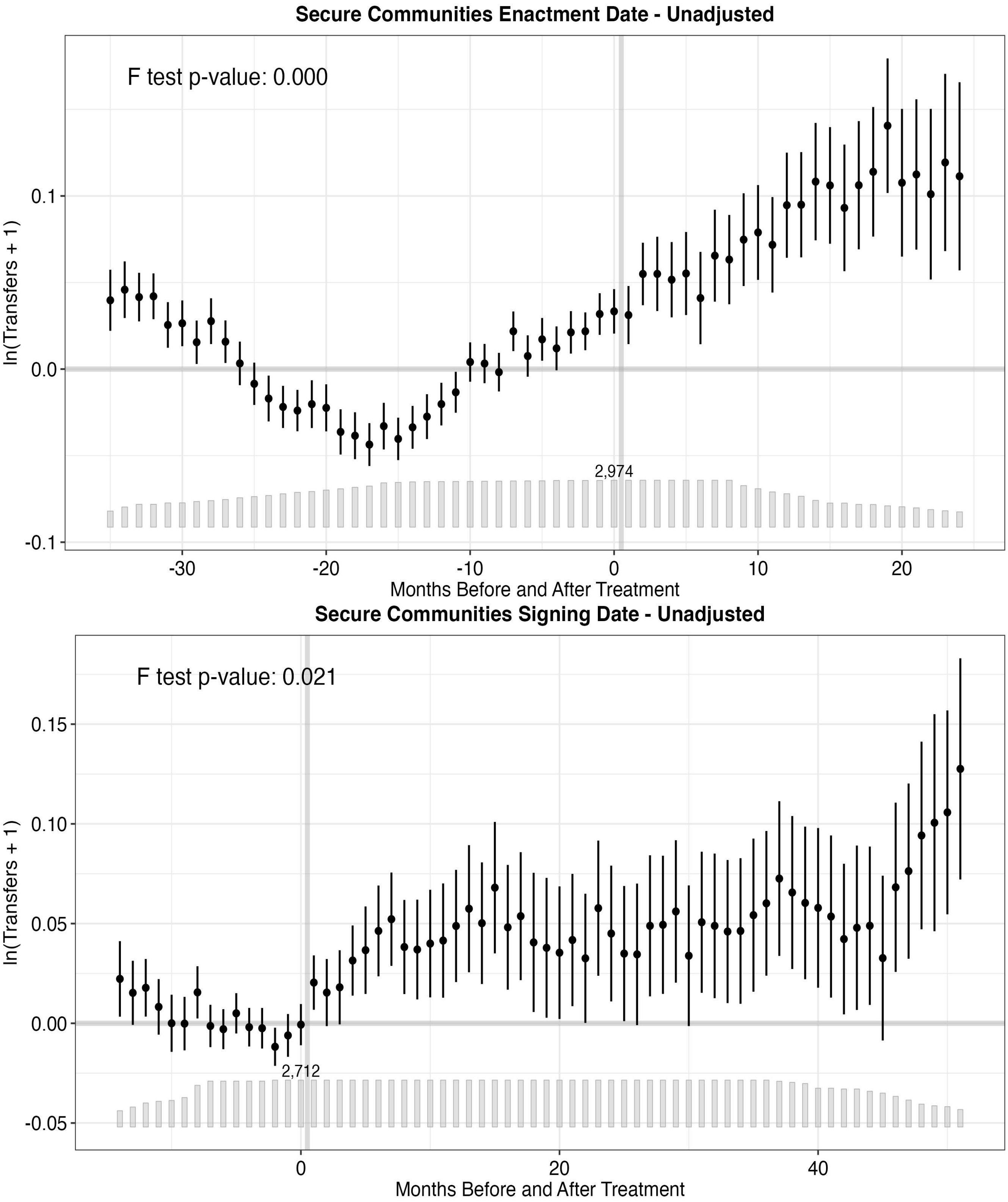

Fig. S15. Impact of Secure Communities Enactment (top) and Signing (bottom) on Counts of Individuals Transferred to ICE custody. The F-test evaluates zero residual averages in the pre-treatment period. A larger F-test p-value suggests a better pre-trend fitting.

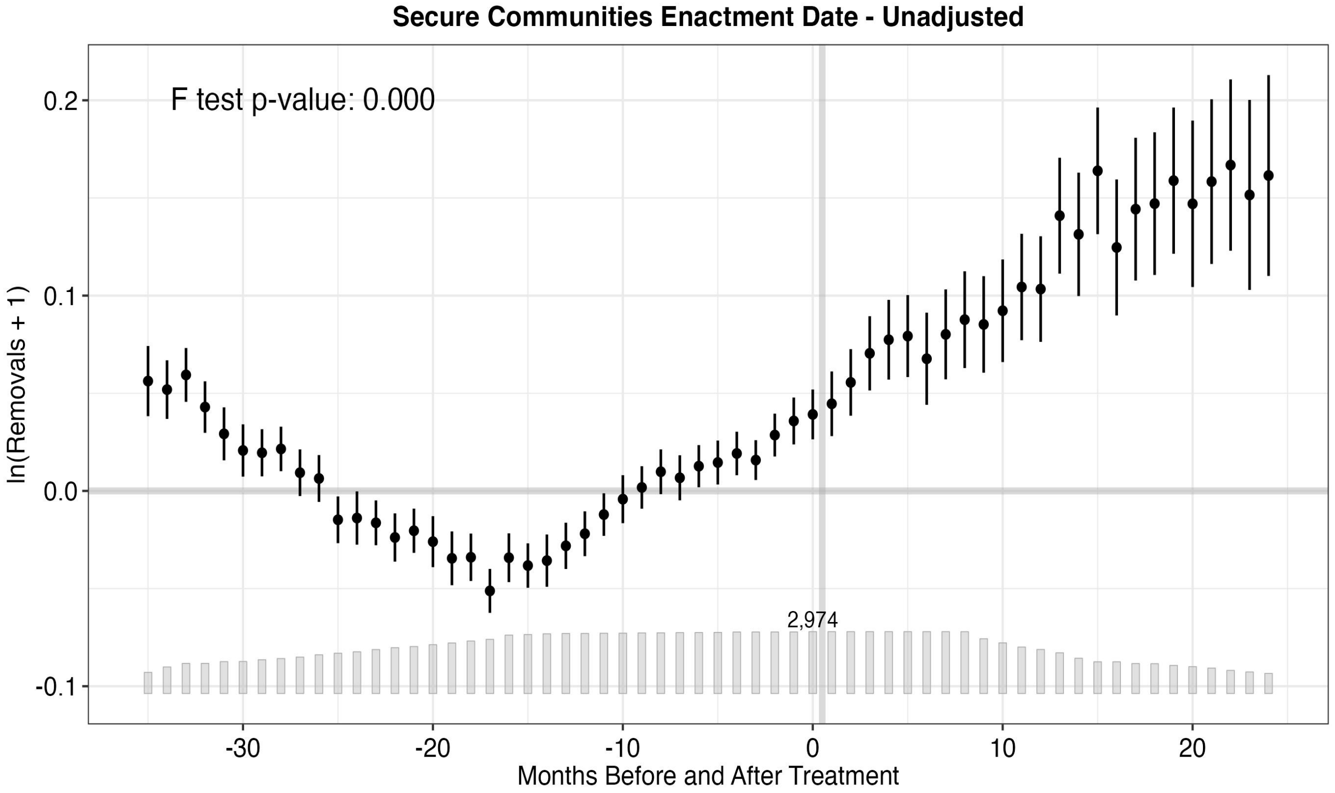

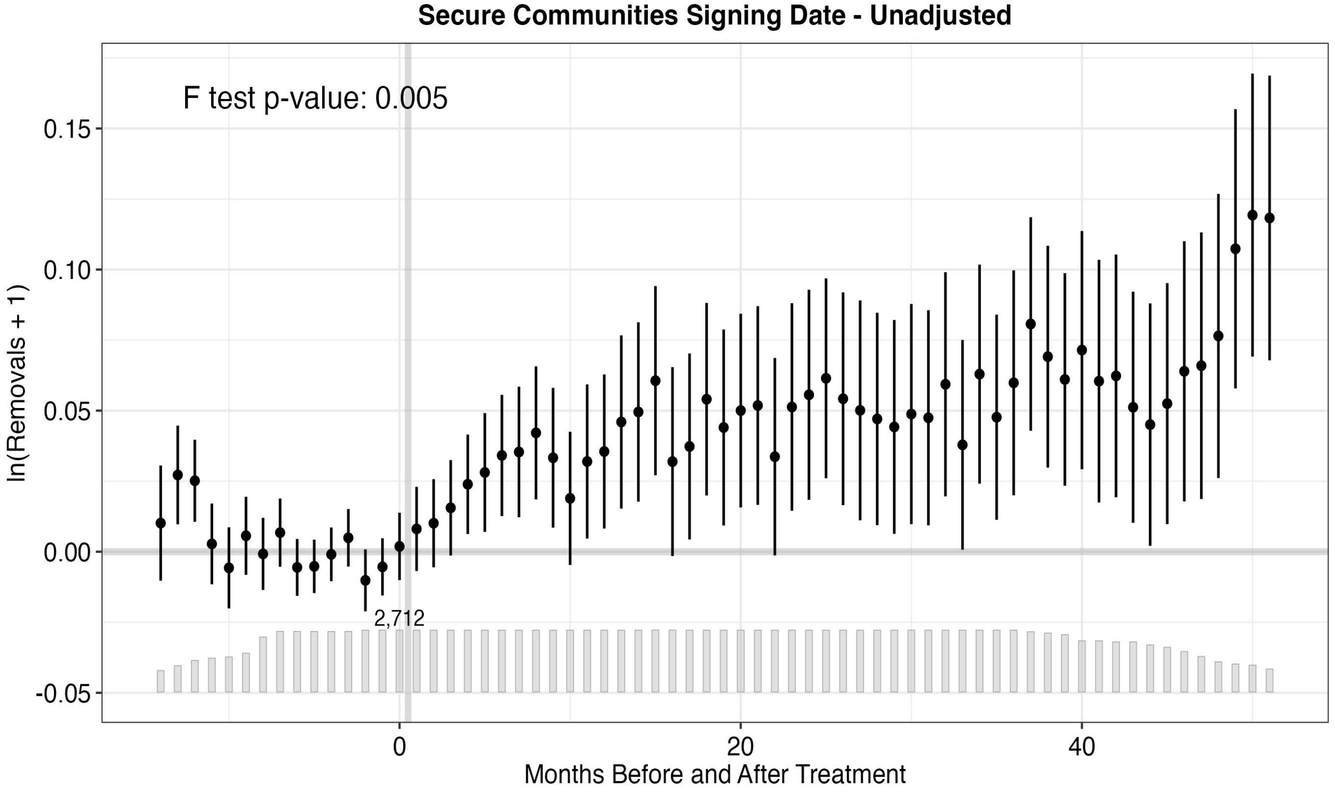

Fig. S16. Impact of Secure Communities Enactment (top) and Signing (bottom) on Counts of Individuals Removed by ICE. The F-test evaluates zero residual averages in the pre-treatment period. A larger F-test p-value suggests a better pre-trend fitting.

Fixed Effects counterfactual estimator (FEct). We relied primarily on the Fixed Effects Counterfactual (FEct) estimator for our results. The Fixed Effects counterfactual (FEct) estimator is an extension of the traditional two-way fixed effect estimator (TWFE).* Generally, the TWFE relies on three assumptions. First, there must be strict exogeneity. That is, the treatment assignment must be independent, as there cannot be anticipation of units self-selecting into the treatment. Showing the presence of parallel trends provides evidence to satisfy this assumption.

Second, there must not be carry over effects or past outcomes that influence the current outcome. Our focus here is on the latter event, given that neither of our treatments "turns off" within the timeframe of our analyses. Figure S17 provides evidence suggesting our data satisfy this assumption. It inquires whether our pre-trends fail a goodness-of-fit test (F Test) or whether the 90% confidence intervals for estimated ATT surpass the equivalence range. The results, with high F test and low equivalence values, suggest pre-trends are not a concern and satisfy the assumption.

Third, the treatment must produce constant treatment effects across units. Compared to traditional TWFE models, however, the FEct estimator allows us to relax the assumption of constant treatment effects. That is, the units need not yield the same effect magnitude from the treatment. Therefore, our results need not have the treatment - signing of an MOA or enactment of Secure Communities - yield the same magnitude across the treated units. Relaxing this assumption helps our inferences; after all, the impact of Secure Communities in San Bernardino, California, likely differs from Lucas County, Ohio. Therefore, relying on FEct allows us to maximize the benefits of TWFE without being hamstrung by the assumption of constant treatment effects.

* See Xu (2017) for more detailed information

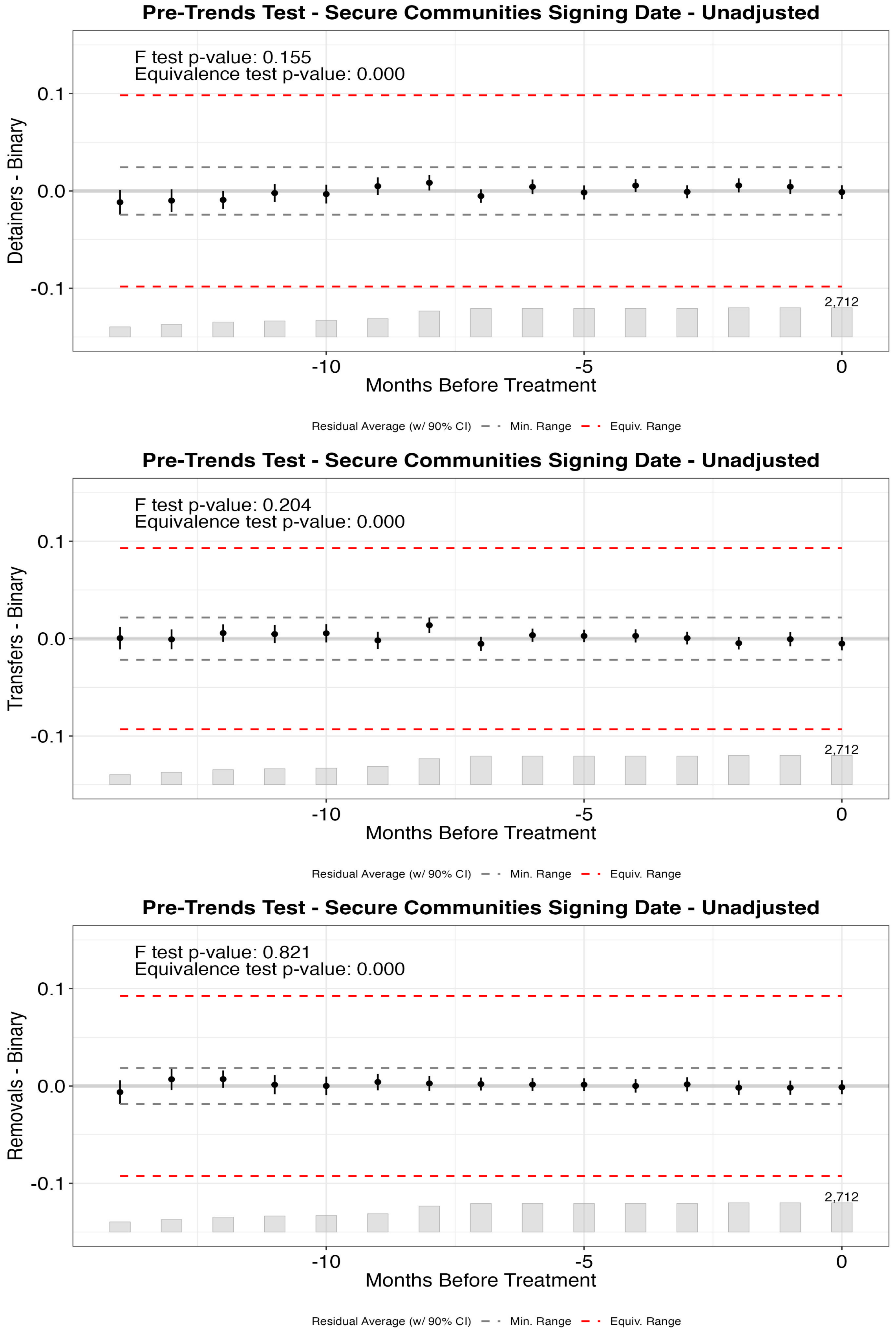

Fig. S17. Test for Pre-Trends Across Detainers (top), Transfers (middle) and Removals (bottom). The F-test evaluates zero residual averages in the pre-treatment period. A larger F-test p-value suggests a better pre-trend fitting. TOST test checks whether the 90% confidence intervals for estimated ATTs in the pre-treatment periods exceed a pre-.36 SD of the outcome. A smaller TOST test p-value suggests a better pre-trend fitting.

Robustness Checks - Alternative Estimators. As an added check, we ran a set of additional estimators. The FEct may be biased if there are underlying time-varying unobserved factors (Xu 2017). If so, our results would not capture the true effect of Secure Communities.

As a robustness check, we also leveraged the Interactive Fixed Effects Counterfactual (IFEct) and Matrix Completion (MC) estimators. To address potential problems, IFEct leverages a factor-augmented models. A necessary step here is choosing the "right" number of factors (r). To avoid handpicking, we allowed the *fect* package to select the ideal number of factors. The process for MC is similar but focuses on hard imputing by selecting a tuning parameter. We follow the same steps and allow the *fect* package to select the best tuning parameter. Figures S18 - S19 present the results from IFEct and MC, respectively. The results remain consistent with those obtained with FEct. In all cases, there is a significant upward trend post-signing of an MOA related to Secure Communities. Critically, the post-treatment change is not accompanied by pre-trends when using signing as a treatment. Therefore, we do not find evidence suggesting that the results are conditional on a specific estimator.

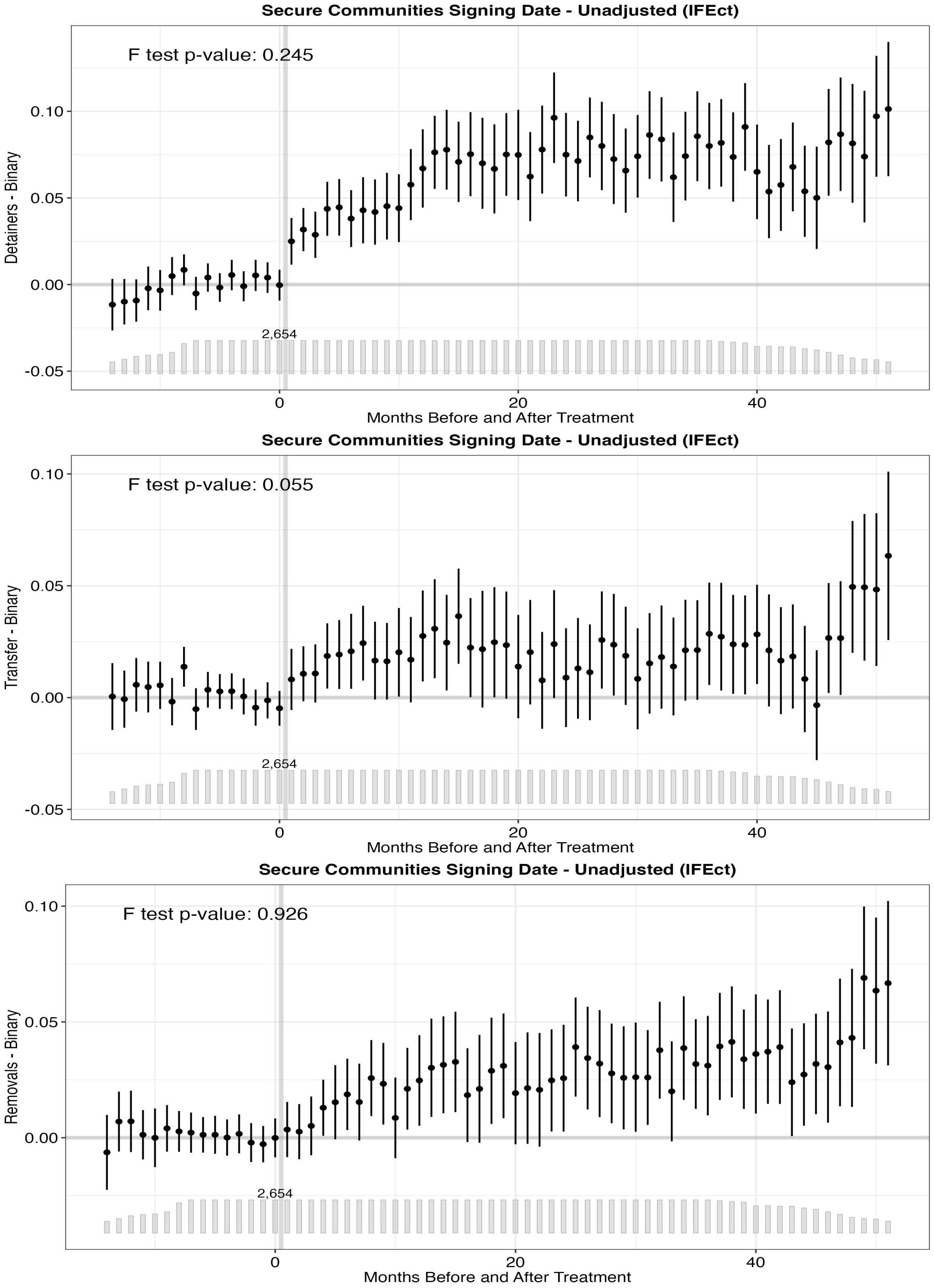

Fig. S18. Impact of Secure Communities Signing on Detainers (top), Transfers (middle), and Removals (bottom). The F-test evaluates zero residual averages in the pre-treatment period. A larger F-test p-value suggests a better pre-trend fitting.

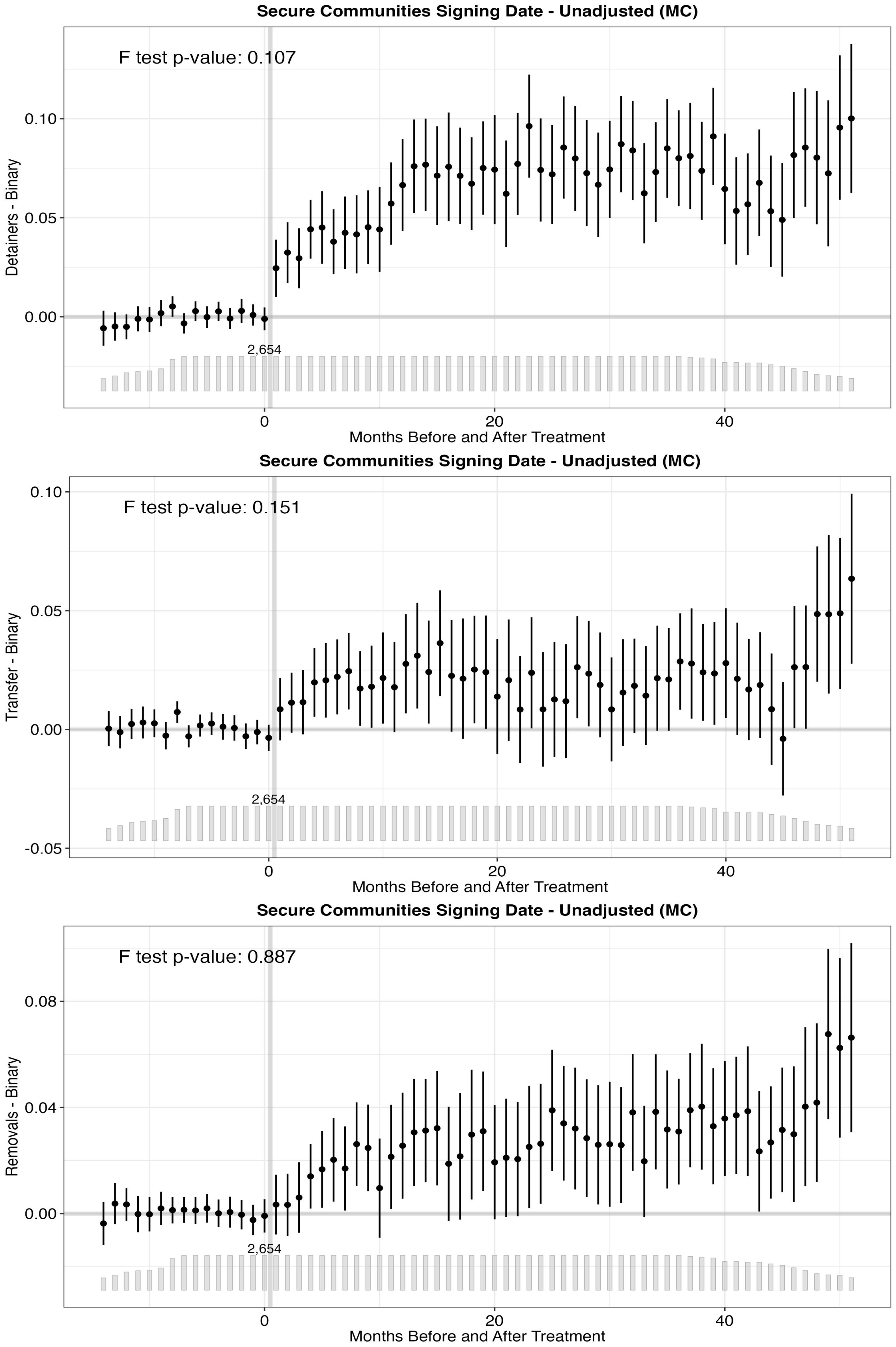

Fig. S19. Impact of Secure Communities Signing on Detainers (top), Transfers (middle), and Removals (bottom). The F-test evaluates zero residual averages in the pre-treatment period. A larger F-test p-value suggests a better pre-trend fitting.

Robustness Checks - Adjusted Results. We examined whether our results reflect unobserved differences in the sociodemographic and sociolegal contexts of our treated and untreated groups. To do so, we estimated a set of adjusted models controlling for seven key variables.

First, we controlled for a county's population by collecting yearly county estimates from the Census. This allows us to account for the possibility that population size may account for any observed differences in detainers. Second, we controlled for the size of the non-citizen population using yearly county data from the American Community Survey. Doing so allows us to control for counties having increased policing given larger populations of non-citizens.

Third, increased ICE enforcement may be (partially) driven by the share of Hispanics in a given county. Therefore, we control for Hispanic (Latino) population by including yearly county estimates collected via the Census. Fourth, lower-income areas may be the target of increased policing. Therefore, we included estimates of the percentage of households below the poverty line from Census data.

Fifth, attitudes about interior immigration enforcement have split across party lines, with Democrats generally in favor and Republicans generally opposed. These political attachments can influence attitudes toward immigration policies - such as Secure Communities - while also fostering an environment that may be more anti-immigrant. Thus, we controlled for local partisan sentiment by leveraging the Republican presidential vote share in the 2008 and 2012 elections. Each county's vote share for the Republican presidential candidate assigned it a specific value that was maintained until the next election (e.g. 2012 election values were assigned for the months in 2012 and 2013). We note that FIPS code 15005, Kalawao County, Hawaii, did not have election data reported. We are grateful for the data made available by the MIT Election Data and Science Lab (2018).

Sixth, police activity may be directly linked with ongoing crime. If so, places with greater crime will likely yield more interactions between police officers and community members. These interactions will produce more fingerprints uploaded to databases, providing greater opportunities for ICE to issue detainers. Therefore, we used the Uniform Crime Reports (UCR) from the FBI to control for the effect of local crime. We are thankful to Kaplan (2021) and the Inter-university Consortium for Political and Social Research (ICPSR) for making this dataset available. We note two changes to these data. First, we collapsed the reporting across agencies into their respective counties. Second, crimes for all five boroughs making up the New York City (NYC) metropolitan area were all assigned to Manhattan. From 2009-2013, the data were also reported in batches with larger-than-expected numbers for January, February, and March, and an even larger-than-expected number for April. We made two assumptions to address this reporting problem. First, we assumed that the relatively large number for April represented crime that occurred during January-March; thus, we added all four months and split the rates evenly. Second, rather than speculate on the crime rate across counties, we assigned each borough a crime rate proportional to its share of the NYC population totals. Therefore, if county X has 10% of the population in NYC, we also assigned it 10% of the crimes. A similar reporting problem affected other counties, and we used similar steps to address misreporting.

Seventh, the rollout of Secure Communities is not the first instance of the federal government seeking to strengthen the communication between police agencies and ICE. Section 287(g) of the Immigration and Nationality Act, rolled out prior to Secure Communities, sought to help identify immigrants by collaborating with officers in jails. Although different in scope and methods than Secure Communities, counties that had participated in 287(g) may have been better prepared and/or more willing to assist ICE in Secure Communities. To account for this possibility, we relied on data on the presence of 287(g) agreements compiled by Asad and Baer-Bositis (2025). They first compiled data from the Urban Institute on 287(g) agreements, which they then verified and corrected by downloading all 287(g) agreements posted by the federal government using the Wayback Machine. We controlled for whether a county had ever been active in 287(g) at some point prior to being enrolled in Secure Communities. About 24.3 percent (n = 755) of counties in our data had a 287(g) agreement prior to enrolling in S-Comm.

Tables S3 and S4, respectively, present the unadjusted and adjusted ATT for signing and enactment on detainers, transfers, and removals. Figures S20 - S25 show the results adjusted for covariates. The results remain consistent in the direction and magnitude as the unadjusted figures in the main text. Therefore, it does not appear that these covariates affect the impact of signing on detainers, transfers, or removals. The results do not suggest that adjusting for covariates addresses the pre-trends affecting using enactment as a treatment. Therefore, something else other than these covariates produces the spike in detainers, transfers, and removals seen in the figures leveraging Secure Communities enactment as a treatment.

	Signing	Enactment
Detainers ATT	6.24	4.93
Detainers 95% CI Low	4.60	3.06
Detainers 95% CI High	7.87	6.80
Transfers ATT	1.81	3.51
Transfers 95% CI Low	0.33	1.74
Transfers 95% CI High	3.28	5.28
Removals ATT	2.36	4.34
Removals 95% CI Low	0.88	2.56
Removals 95% CI High	3.84	6.11

Table S3. Unadjusted ATT of Signing and Enactment Dates on Percentage Point Change in the Probability of Detainers, Transfers, and Removals, 2009-2013

	Signing	Enactment
Detainers ATT	5.99	4.33
Detainers 95% CI Low	4.30	2.50
Detainers 95% CI High	7.68	6.15
Transfers ATT	1.58	2.77
Transfers 95% CI Low	0.06	0.95
Transfers 95% CI High	3.11	4.59
Removals ATT	2.19	3.33
Removals 95% CI Low	0.65	1.48
Removals 95% CI High	3.73	5.19

Table S4. Adjusted ATT of Signing and Enactment Dates on Detainers, Transfers, and Removals, 2009-2013

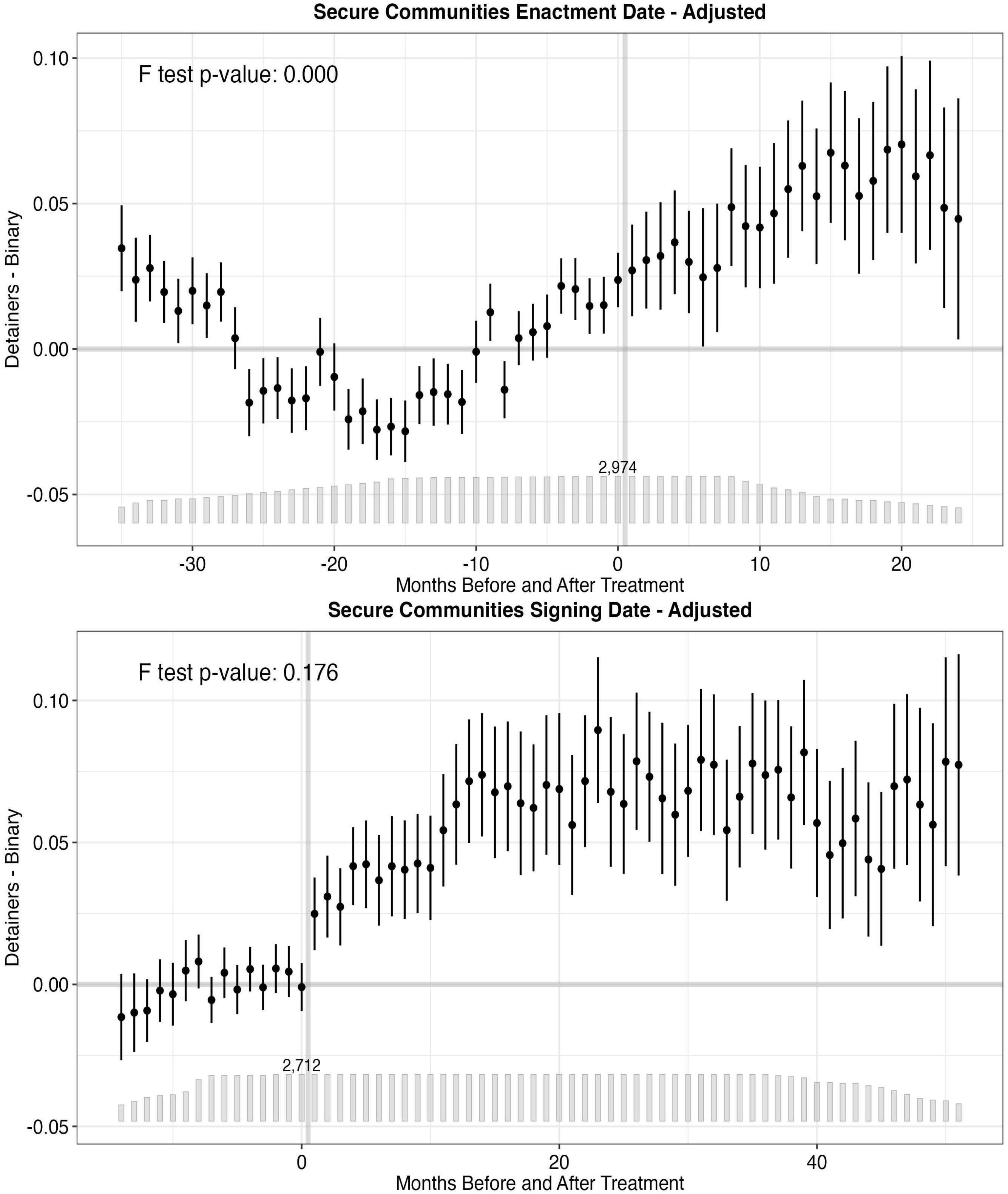

Fig. S20. Impact of Secure Communities Enactment (top) and Signing (bottom) on Detainers issued by ICE. The number within the figure indicates the total treated units (counties). The F-test evaluates zero residual averages in the pre-treatment period. A larger F-test p-value suggests a better pre-trend fitting.

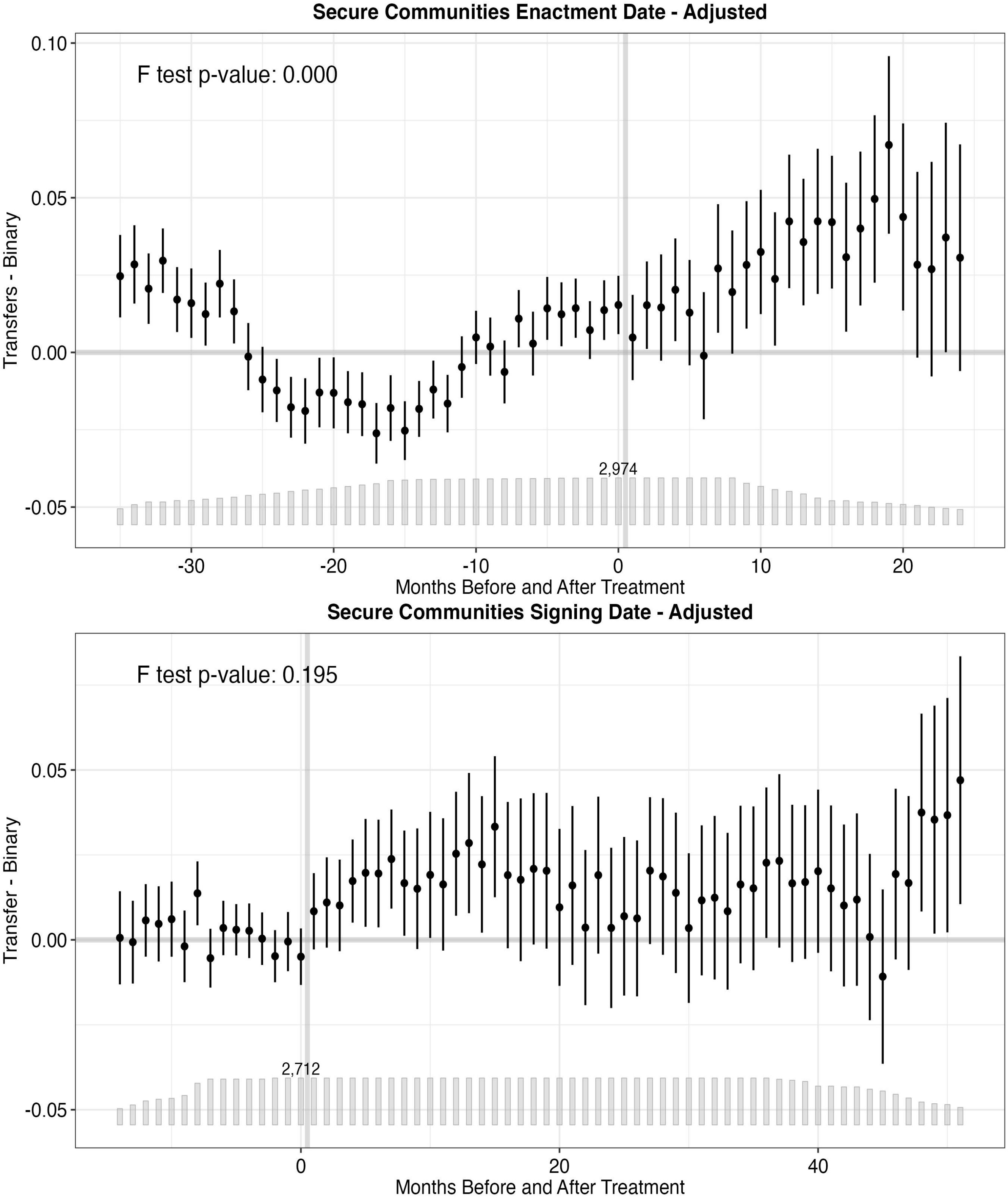

Fig. S21. Impact of Secure Communities Enactment (top) and Signing (bottom) on Transfers to ICE custody. The number within the figure indicates the total treated units (counties). The F-test evaluates zero residual averages in the pre-treatment period. A larger F-test p-value suggests a better pre-trend fitting.

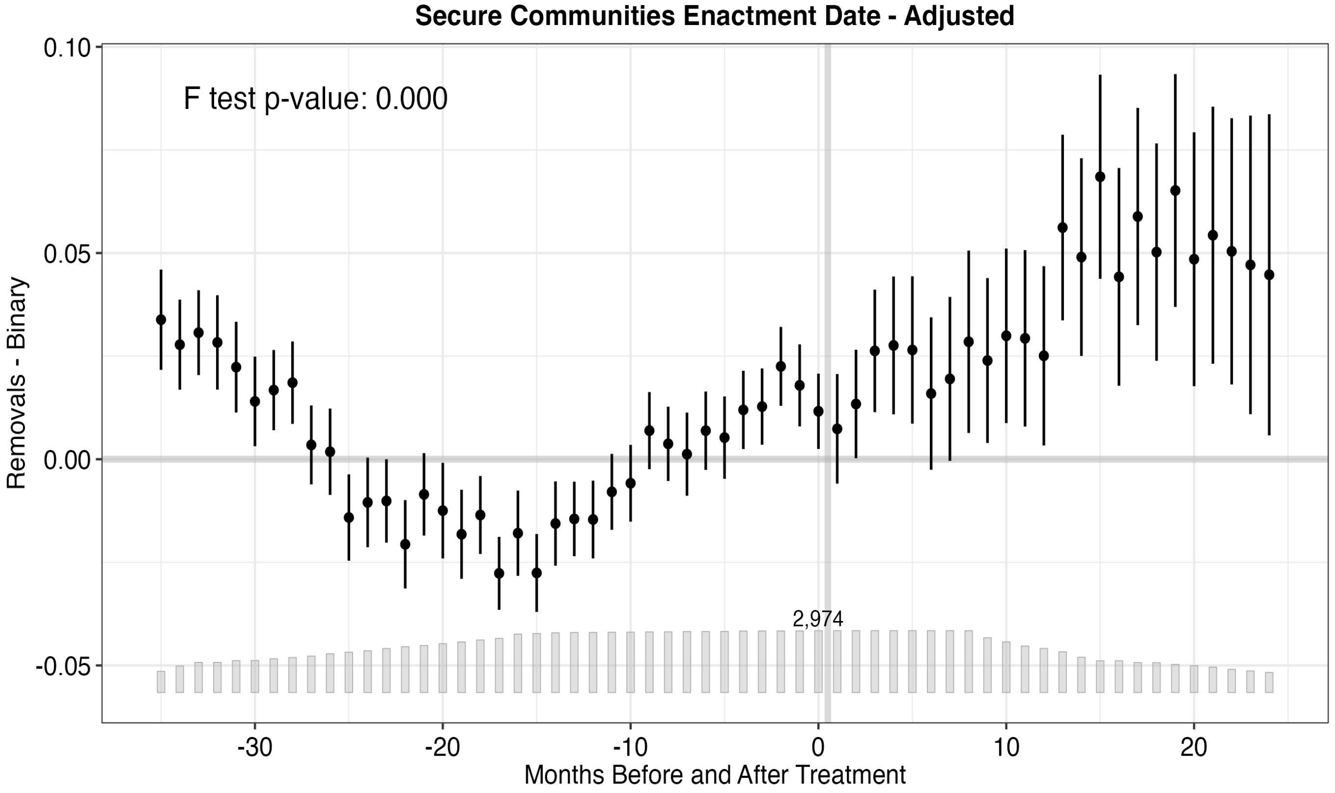

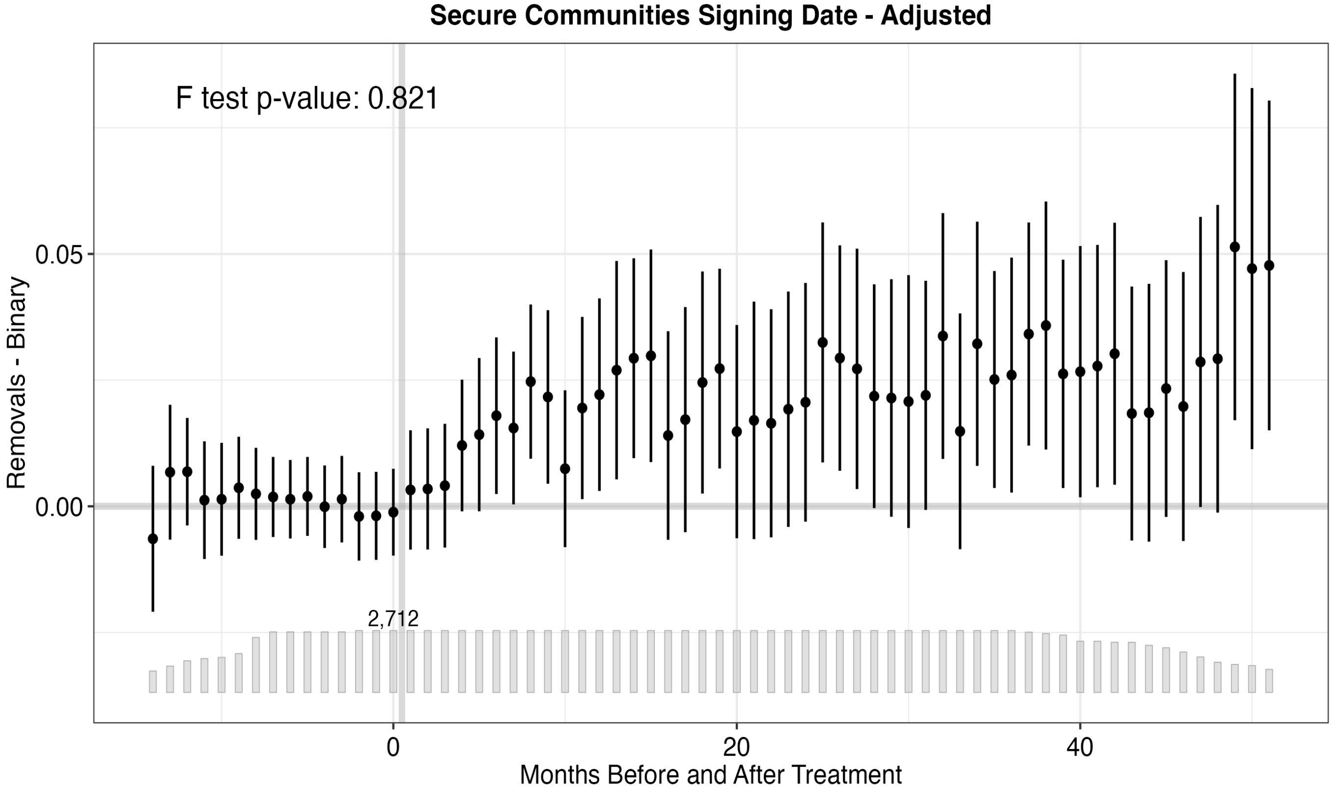

Fig. S22. Impact of Secure Communities Enactment (top) and Signing (bottom) on Removals issued by ICE. The number within the figure indicates the total treated units (counties). The F-test evaluates zero residual averages in the pre-treatment period. A larger F-test p-value suggests a better pre-trend fitting.

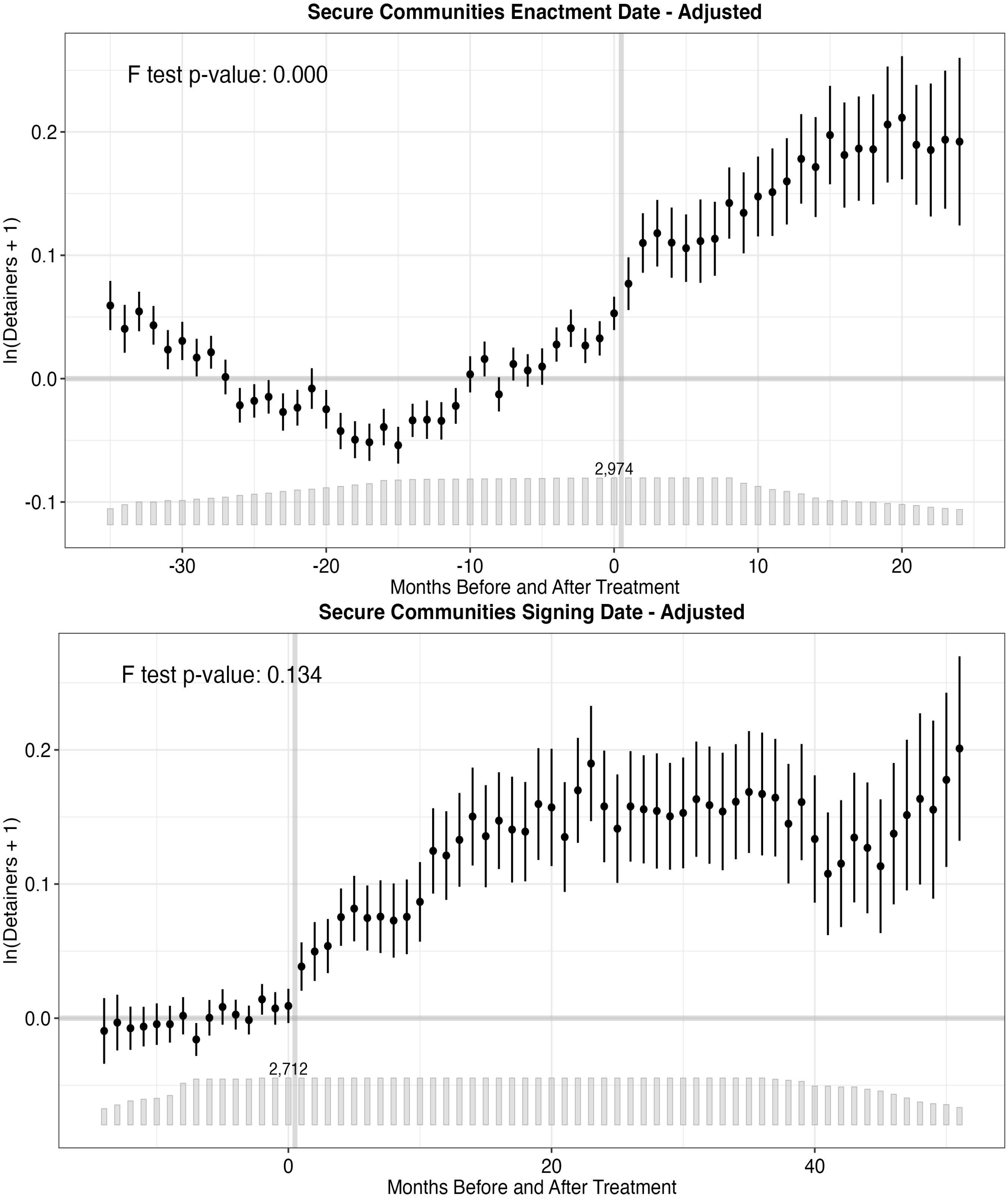

Fig. S23. Impact of Secure Communities Enactment (top) and Signing (bottom) on Detainers issued by ICE. The number within the figure indicates the total treated units (counties). The F-test evaluates zero residual averages in the pre-treatment period. A larger F-test p-value suggests a better pre-trend fitting.

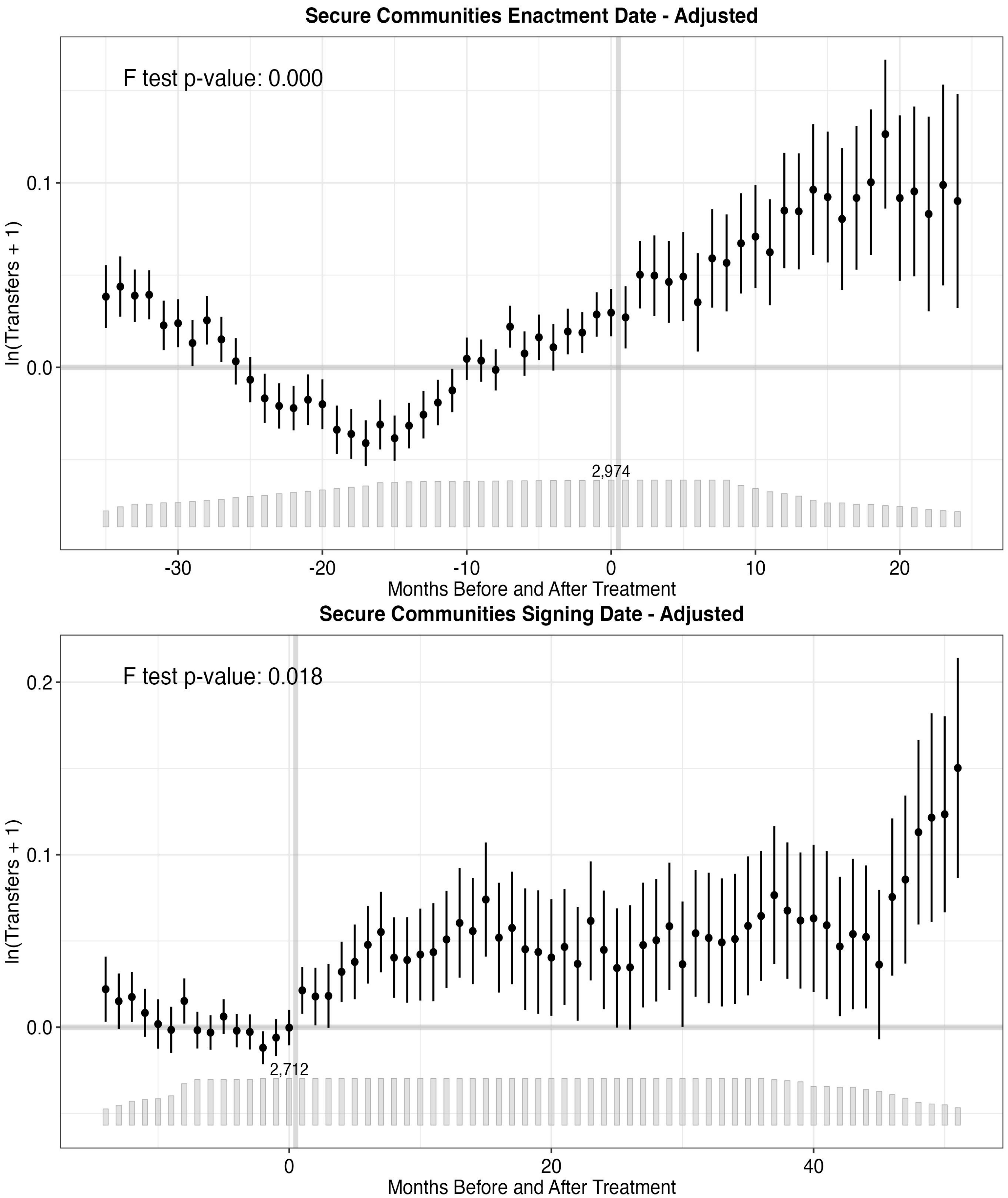

Fig. S24. Impact of Secure Communities Enactment (top) and Signing (bottom) on Counts of Individuals Transferred to ICE custody. The F-test evaluates zero residual averages in the pre-treatment period. A larger F-test p-value suggests a better pre-trend fitting.

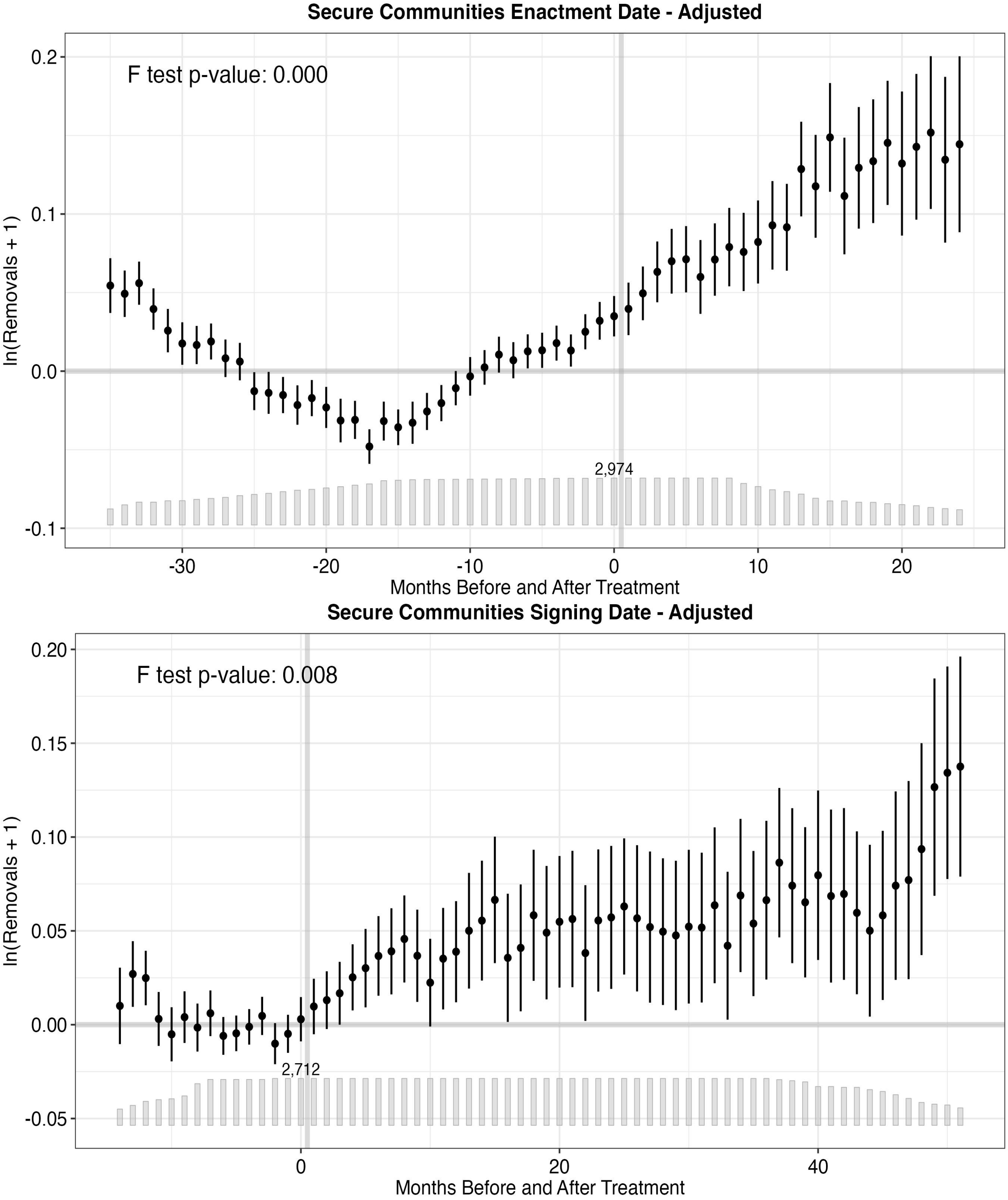

Fig. S25. Impact of Secure Communities Enactment (top) and Signing (bottom) on Counts of Individuals Removed by ICE. The F-test evaluates zero residual averages in the pre-treatment period. A larger F-test p-value suggests a better pre-trend fitting.

Robustness Checks - Enactment and Signing Timing. There are 90 counties that enacted Secure Communities prior to their state signing an MOA. The role of these 90 counties matters for our inference. After all, counties that enacted before their state signed an MOA may differ in significant ways from those that waited. For instance, counties that enacted pre-signing could be more prepared to initiate Secure Communities, hold a strategic role in DHS plans, and/or have more individuals targeted by DHS. Therefore, we excluded these 90 counties. If these counties are driving our results, we should expect the results to differ substantively and/or in terms of statistical significance once they are removed. Figure S26 presents the results after dropping the 90 counties. Overall, the results remain consistent with the main results. The average unadjusted ATT using FEct for detainers is 0.049 [0.031,0.067], transfers is 0.030 [0.012,0.048], and removals 0.016 [-0.0006,0.032].

One potential concern with the results that exclude the 90 counties in which enactment preceded a state's signing of an MOA is the F-test. In all plots we present, the F-test evaluates zero residual averages in the pre-treatment period. A larger F-test p-value suggests a better pre-trend fitting. Across all three plots in Figure S26, we reject the null hypothesis of zero residual averages in the pre-treatment period; this indicates the possibility of a pre-trend.

To further investigate the possible pre-trend, we conducted a two-one-sided t (TOST) test, a type of equivalence test. The TOST test checks whether the 90% confidence intervals for estimated ATTs in the pre-treatment periods exceed a pre-specified range (here, 0.36 standard deviations of the outcome variable), or the equivalence range. A smaller TOST test p-value suggests a better pre-trend fitting. The TOST equivalence tests presented in Figure - S27 do not allow us to reject the null hypothesis, which indicates that the 90% confidence intervals do not exceed this pre-specified range; this indicates that any identified pre-trend is not substantively meaningful. Taken together, we believe both the F-test and the TOST test support our interpretation that signing dates are consequential for increases in detainers, transfers, and removals.

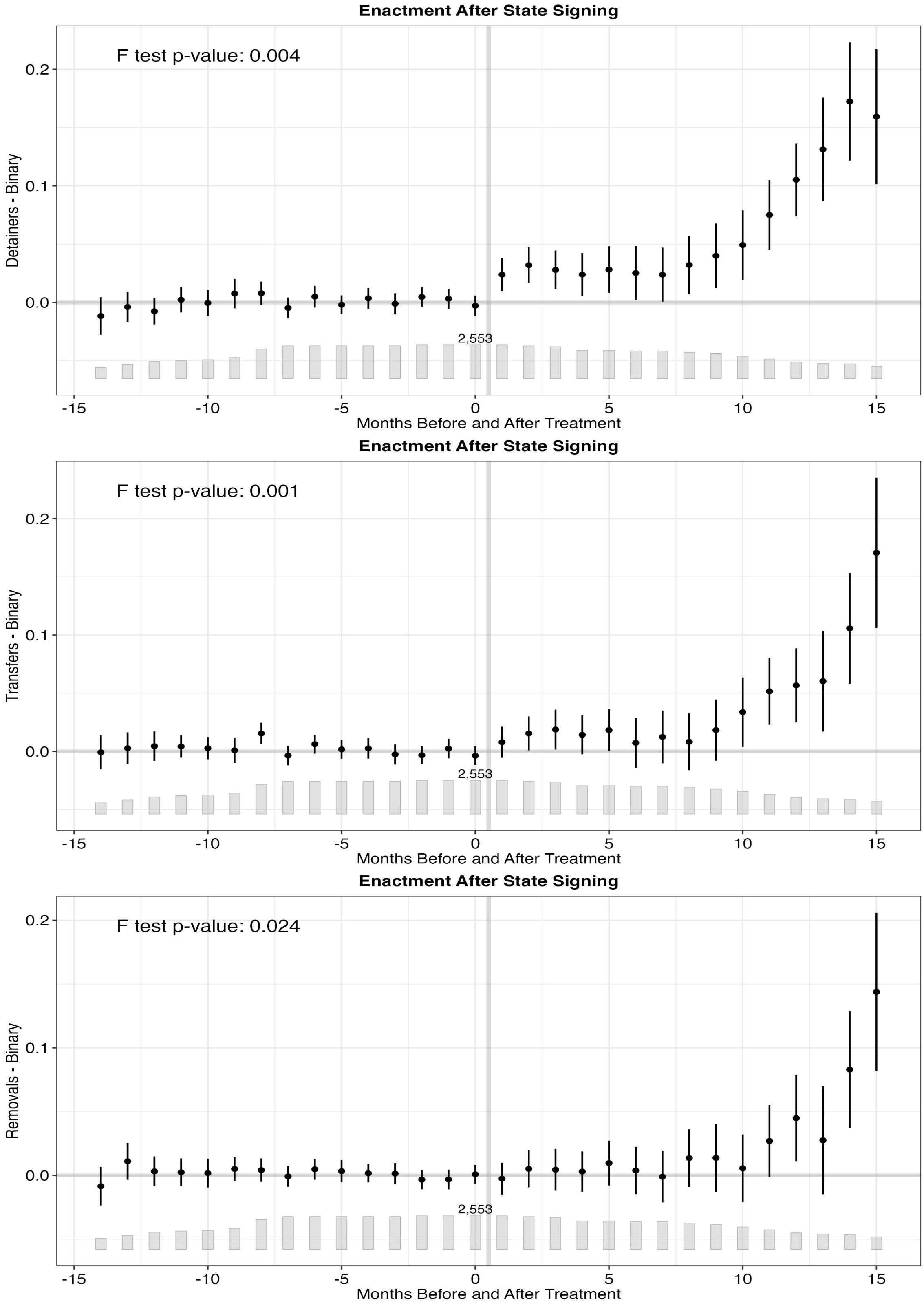

Fig. S26. Impact of Secure Communities MOA Signing on Detainers (top), Transfers (middle), and Removals (bottom) among Counties that Enacted Secure Communities After their State Signed an MOA. The F-test evaluates zero residual averages in the pre-treatment period. A larger F-test p-value suggests a better pre-trend fitting.

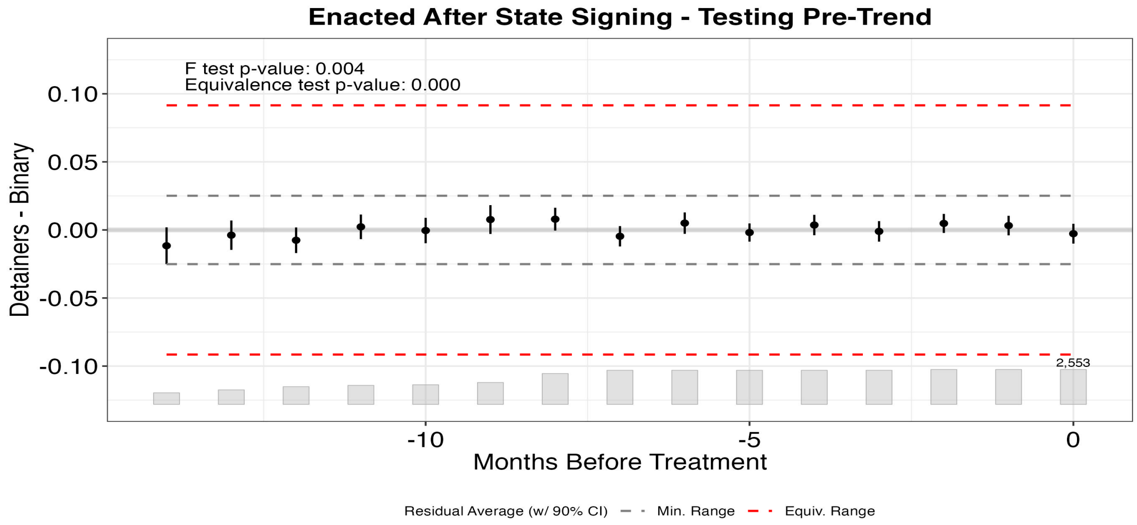

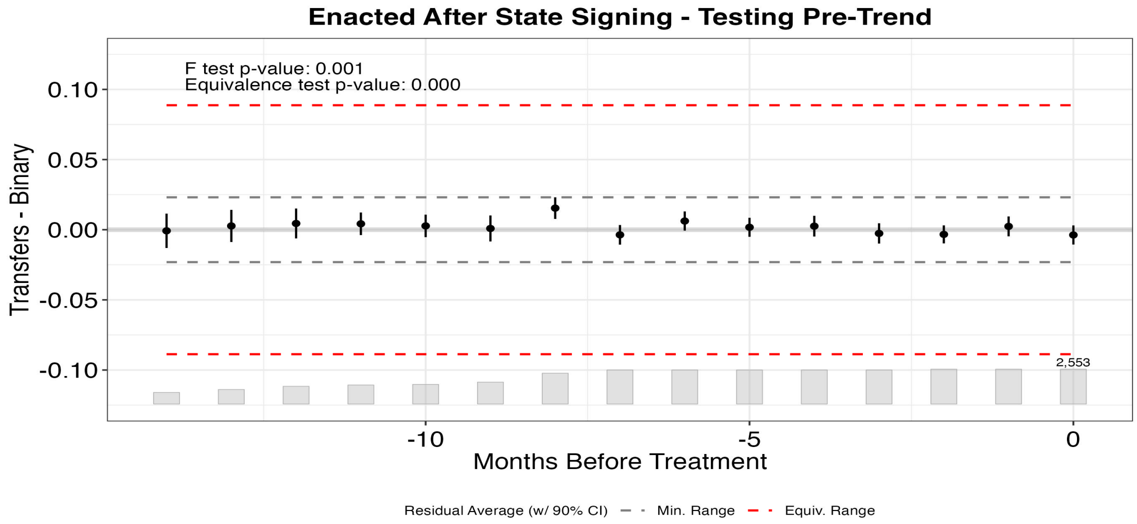

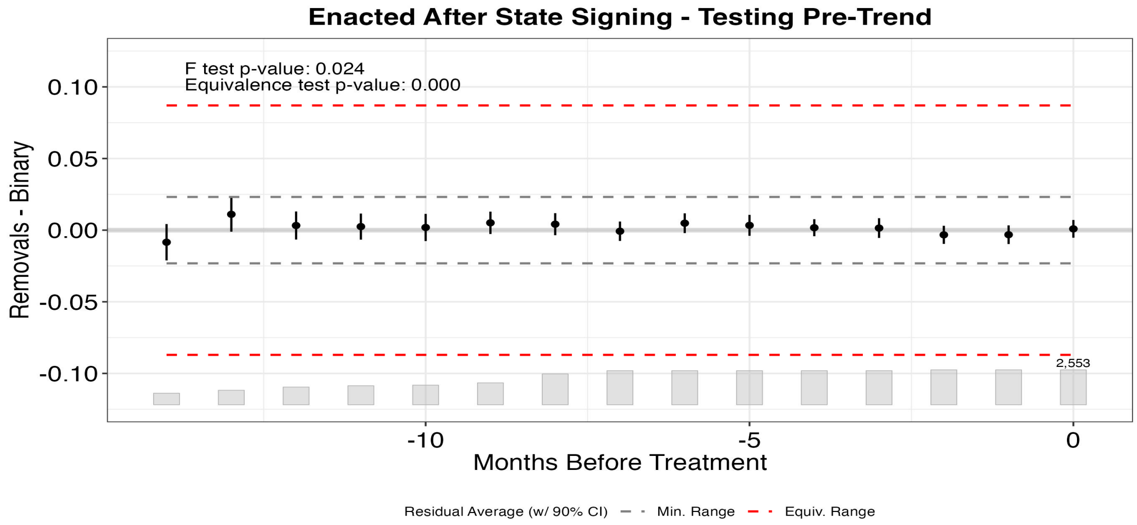

Fig. S27. Two Tests for Pre-Trends in Detainers (top), Transfers (middle), and Removals (bottom) Outcomes among Counties that Enacted Secure Communities After their State Signed an MOA, 2009-2013. The F-test evaluates zero residual averages in the pre-treatment period. A larger F-test p-value suggests a better pre-trend fitting. TOST test checks whether the 90% confidence intervals for estimated ATTs in the pre-treatment periods exceed a pre-.36 SD of the outcome. A smaller TOST test p-value suggests a better pre-trend fitting.

Robustness Checks - Clustered SEs. Given the nature of our data, it is also important to explore whether state-level factors influenced our treatment. To do so, we re-estimated our results including state-level clustered standard errors. Figures S28 and S29 present the unadjusted and adjusted results, respectively. The unadjusted ATT with cluster-level standard errors for detainers is 6.24 [-2.48,14.95], transfers is 1.81 [-4.43,8.04], and removals is 2.4 [-3.43, 8.15]. When including covariates, the adjusted ATT for detainers is 5.98 [-2.92,14.88], transfers is 1.58 [-4.80, 7.96], and removals is 2.18 [-3.83, 8.19].

We note that, while the substantive interpretation of our results is consistent, they no longer reach conventional levels of statistical significance. Despite the loss of significance, there are key reasons for which we believe the results remain important. Clustering at the state level is not a straightforward process in our case. To be sure, past scholarship such as Abadie et al. (2023) recommend that "standard errors should be clustered at the treatment assignment level." But in our case, this advice is less straightforward. That is, to our knowledge, nothing is occurring policy-wise across the whole state when they sign an MOA. Rather, the state is signaling that it will be fully compliant with Secure Communities–even if its counties have not yet activated. Put differently, a state signing an MOA at time t indicates their counties will enroll at a later $t+1$ date. The MOA, however, does not preclude counties from enacting Secure Communities anytime before t+1 occurs. Indeed, as presented in Figures S2 - S3, there is significant variation in the timing of enactment and signing.

	ATT	95% CI Low	95% CI High
Detainers - Unadjusted	6.24	-2.48	14.95
Transfers - Unadjusted	1.81	-4.43	8.04
Removals - Unadjusted	2.36	-3.43	8.15
Detainers - Adjusted	5.98	-2.92	14.88
Transfers - Adjusted	1.58	-4.80	7.96
Removals - Adjusted	2.18	-3.83	8.19

Table S5. Impact of Signing Dates on County Detainers, Transfers, and Removals with State-level Clustered Standard Errors, Adjusted and Unadjusted

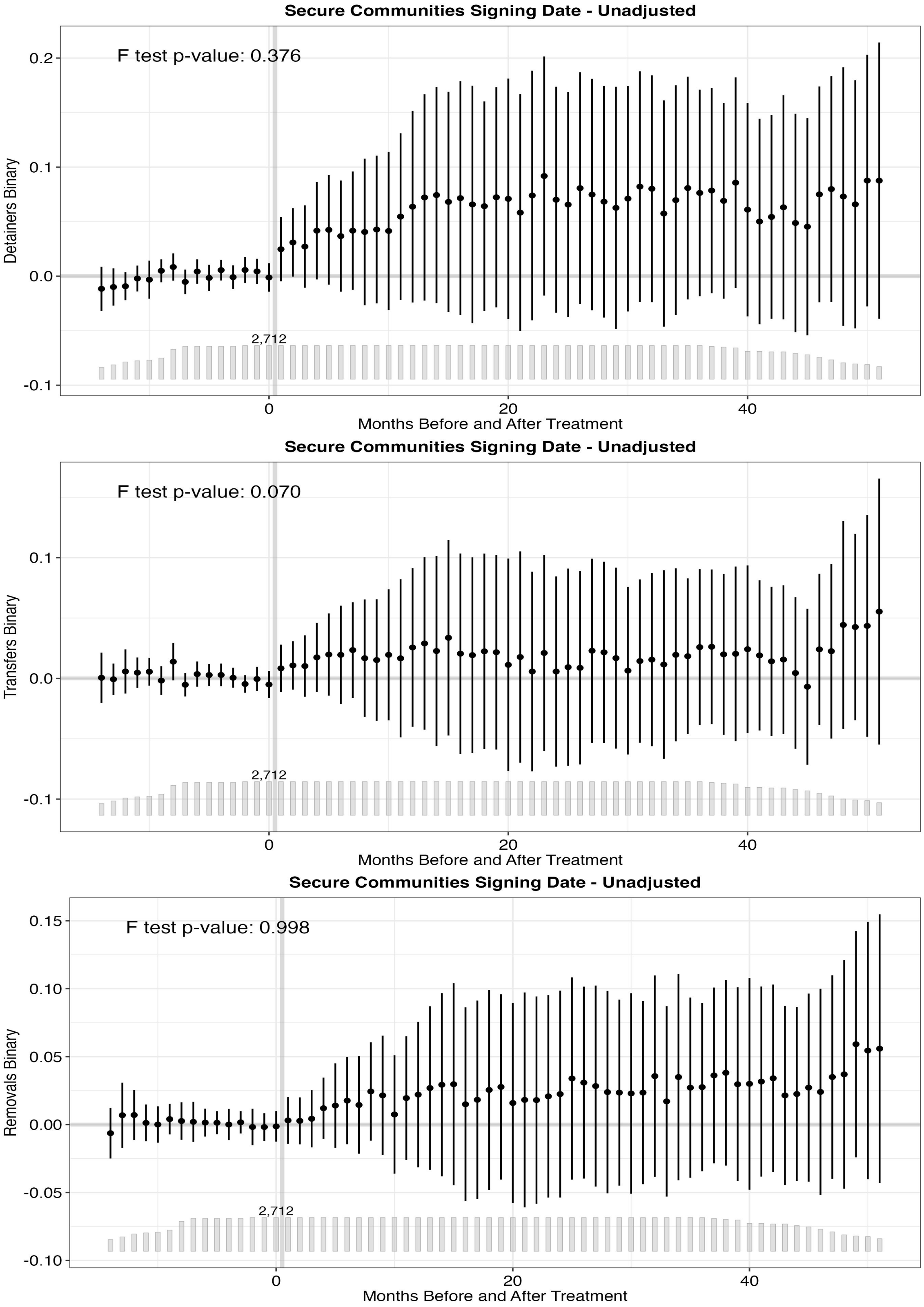

Fig. S28. Impact of Secure Communities Signing on Detainers (top), Transfers (middle), and Removals (bottom) with State-Level Clustered Standard Errors. The F-test evaluates zero residual averages in the pretreatment period. A larger F-test p-value suggests a better pre-trend fitting.

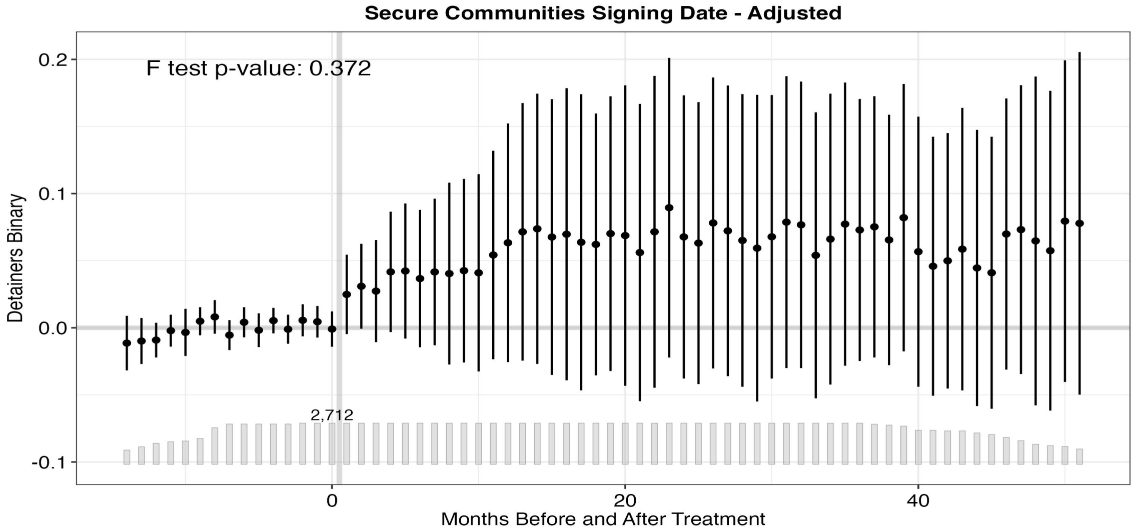

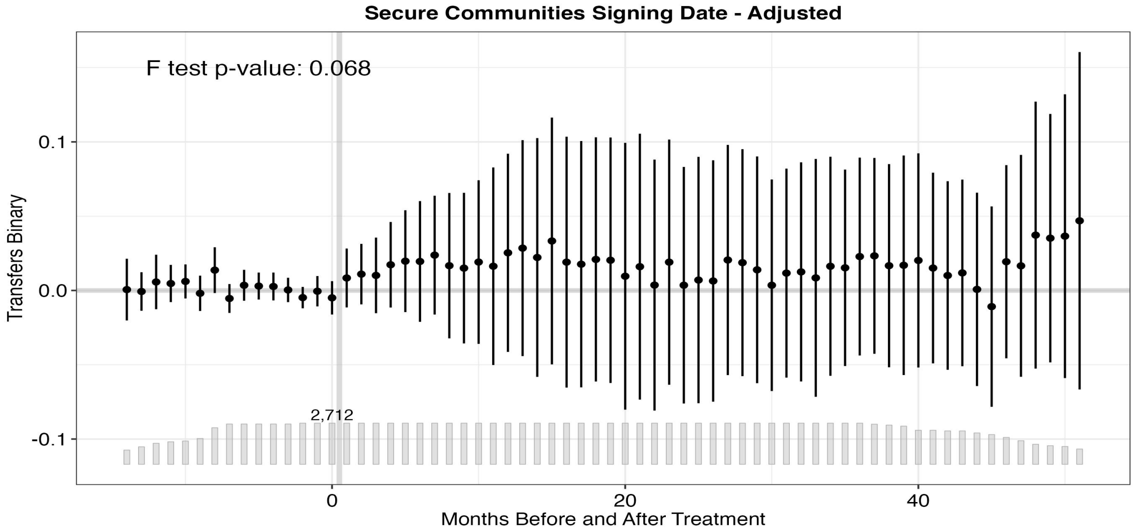

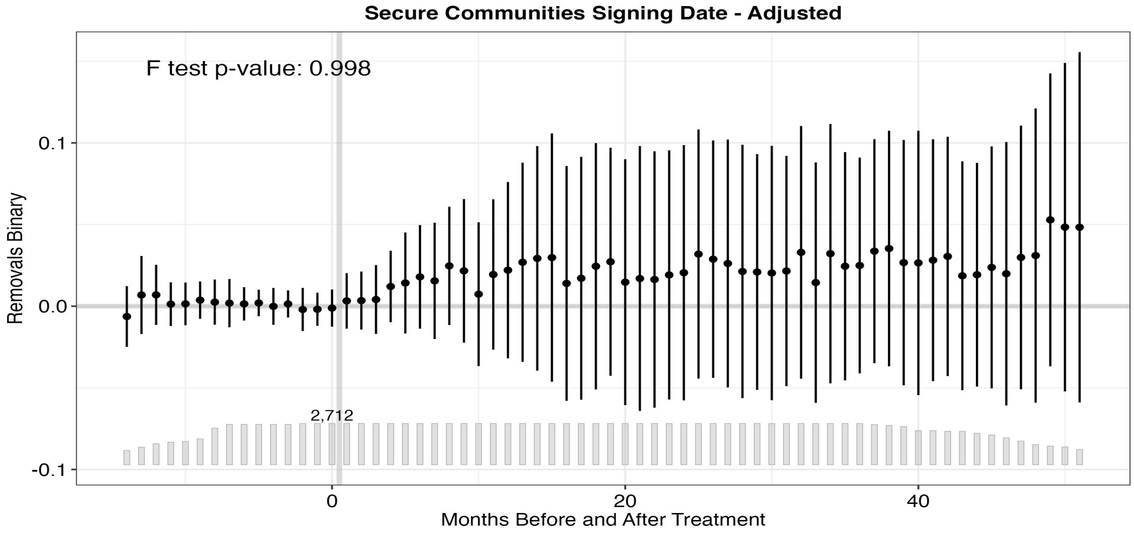

Fig. S29. Impact of Secure Communities Signing on Detainers (top), Transfers (middle), and Removals (bottom) with State-Level Clustered Standard Errors. The F-test evaluates zero residual averages in the pretreatment period. A larger F-test p-value suggests a better pre-trend fitting.

Robustness Checks - 287(g). Secure Communities was not the first attempt at strengthening the communication between local police agencies and federal immigration officers. Figure S30 shows the locales that, at some point, had enacted 287(g) agreements.

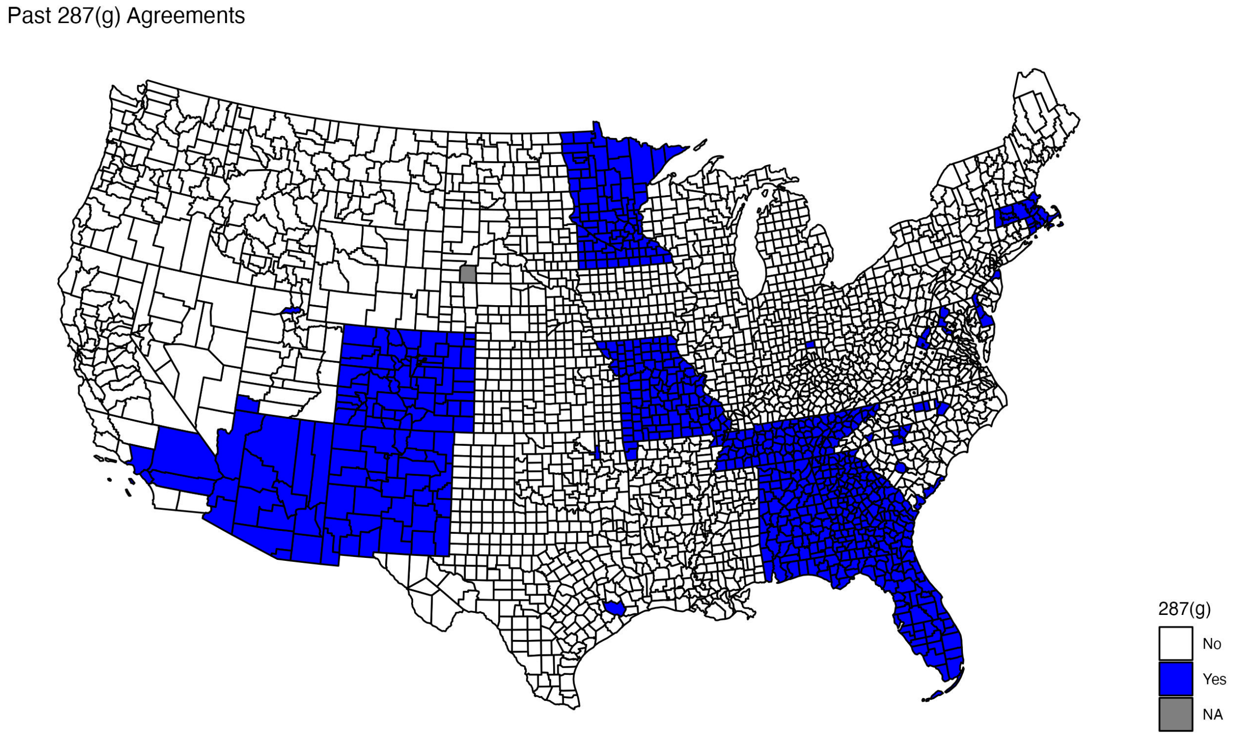

Fig. S30. Counties with History of 287(g) Agreements

Given the historical role of 287(g) agreements, we investigated whether its presence influenced our results. We do so in two ways. First, we controlled for past presence of 287(g) agreements in adjusted models. Figures S20 - S22 show the impact of signing Secure Communities MOAs after adjusting for past history of 287(g) and other key variables. The figures show results consistent with the unadjusted models.

Second, we analyze separately counties with and without past 287(g) agreements. Table 1 in the main text and Table S6 here show the unadjusted and adjusted results, respectively, from counties in states that signed an MOA with and without past histories of 287(g) agreements. The results show a past history of 287(g) agreements is a primary driver of detainer issuance following a state's signing of an MOA pertaining to Secure Communities. The results, however, show null effects in transfers and removals in counties whose states sign an MOA for Secure Communities but lack a prior history of 287(g) agreements.

A few factors could explain these results. First, counties with 287(g) agreements may have enjoyed a logistical advantage. That is, prior experience with the institutional precursor to Secure Communities may have prepared these counties to better communicate with ICE. Similarly, ICE could have gained valuable knowledge for assisting them in their immigration-enforcement efforts. If so, previous 287(g) experience could have provided a practice run aiding ICE once their states signed a Secure Communities MOA. The evidence we present of a prior 287(g) agreement's effects on transfers and removals aligns with our interpretation that a prior collaboration between local police and federal immigration authorities facilitated the transfer of noncitizens into ICE custody for eventual removal, though it does not directly confirm it.

Second, counties with past 287(g) agreements may have a higher concentration of noncitizens. The map in Figure S30 shows that counties with past 287(g) agreements are present in key border states such as California, Arizona, New Mexico, and Florida. Therefore, the results in the tables may reflect the presence of a larger pool of individuals eligible for transfer and removal. The results provide some limited evidence for both possibilities. The results show positive and significant effects of a prior 287(g) agreement on detainers following the signing of Secure Communities. Therefore, ICE also issued more detainers after their state signed an MOA for Secure Communities. But as the lower two panels show, the higher number of detainers did not ultimately lead to more transfers or removals from the country.

Exploring the underlying mechanisms of the relationship of past histories of 287(g) and the impact of Secure Communities requires a much more in-depth analysis. Doing so, unfortunately, is outside the scope of this paper. Independent of the underlying factors, however, the results in the tables show variation in the impact of signing across counties. Therefore, future scholarship should explore the role of varying anti-immigration policies over time, even as some end or evolve. Perhaps the memory of these policies leaves an imprint that can influence the impact of policies enacted at a later date.

	Overall	287(g) YES	287(g) NO
Detainers ATT	5.99	11.40	4.65
Detainers 95% CI Low	4.30	8.06	2.65
Detainers 95% CI High	7.68	14.74	6.65
Transfers ATT	1.58	5.60	0.52
Transfers 95% CI Low	0.06	2.28	-1.28
Transfers 95% CI High	3.11	8.92	2.33
Removals ATT	2.19	5.20	1.42
Removals 95% CI Low	0.65	1.93	-0.42
Removals 95% CI High	3.73	8.48	3.27

Table S6. Adjusted ATT of MOA Signing Date on Counties with and without Prior 287(g) Agreements

Data Sources

We compiled various data sources to create an original dataset that allowed us to investigate the impact of Secure Communities on total detainers, transfers, and removals. Below we describe the process to merge these different datasets together.

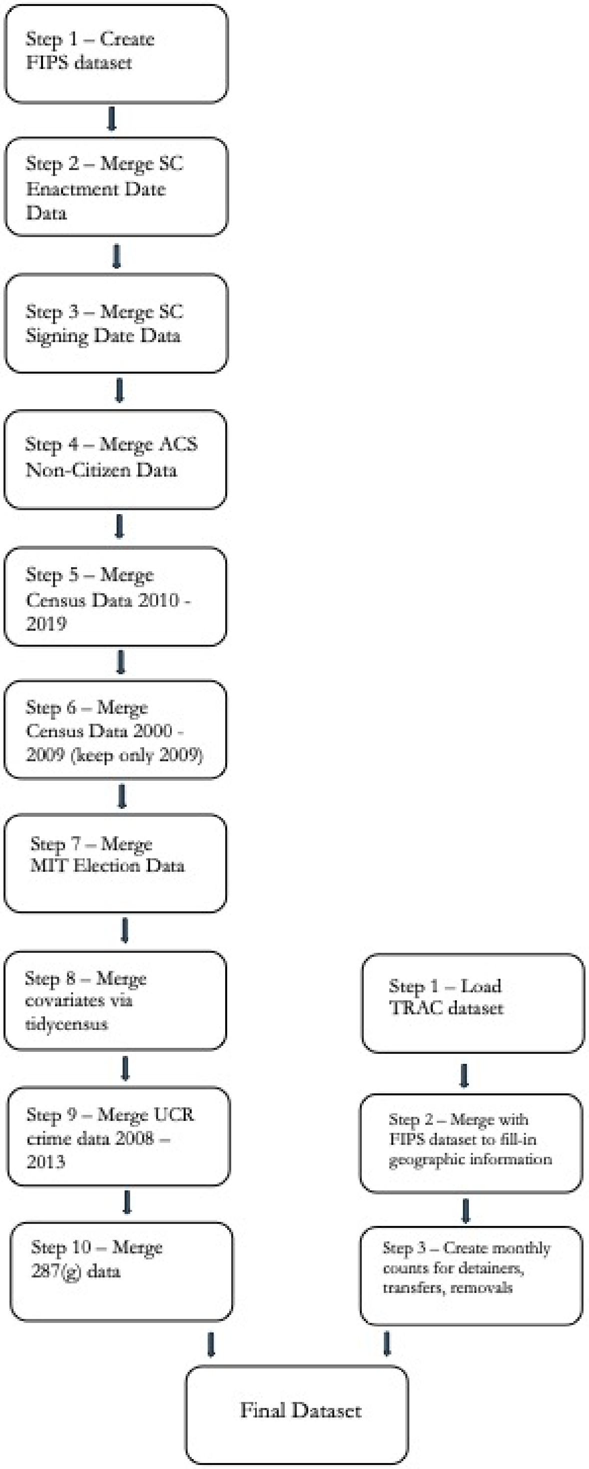

Fig. S31. Flowchart Outlining the Process to Create the Final Dataset used in Analyses

References

1. Abadie, Alberto, Susan Athey, Guido W. Imbens, and Jeffrey M. Wooldridge. "When should you adjust standard errors for clustering?." The Quarterly Journal of Economics 138, no. 1 (2023): 1-35.

2. Asad, Asad L., and Livia Baer-Bositis. 2025. “Spatial and Temporal Contexts of Formal Social Control and System Involvement: U.S. Latinos under Immigration Policing.” Law & Society Review 59 (1): 172-208.

3. Chen, Jiafeng, and Jonathan Roth. 2024. "Logs with Zeros? Some Problems and Solutions." The Quarterly Journal of Economics 139 (2): 891-936.

4. Kaplan, Jacob. 2021. "Jacob Kaplan's Concatenated Files: Uniform Crime Reporting Program Data: Offenses Known and Clearances by Arrest (Return a), 1960-2020." Ann Arbor, MI: Inter-university Consortium for Political and Social Research.

5. MIT Election Data and Science Lab. 2018. "County Presidential Election Returns 2000-2020." Harvard Dataverse, V13.

6. White, Ariel. 2016. "When Threat Mobilizes: Immigration Enforcement and Latino Voter Turnout." Political Behavior 38: 355-382.

7. Xu, Yiqing. 2017. "Generalized synthetic control method: Causal inference with interactive fixed effects models." Political Analysis 25.1: 57-76.
